# Clinimetric properties of lower limb neurological impairment tests for children and young people with a neurological condition: A systematic review

**DOI:** 10.1371/journal.pone.0180031

**Published:** 2017-07-03

**Authors:** Ramona Clark, Melissa Locke, Bridget Hill, Cherie Wells, Andrea Bialocerkowski

**Affiliations:** 1 Menzies Health Institute Queensland, Griffith University, Gold Coast, Queensland, Australia; 2 School of Allied Health Sciences, Griffith University, Gold Coast, Queensland, Australia; 3 Movement Solutions, Coorparoo, Queensland, Australia; 4 Epworth Monash Rehabilitation Medicine Unit, Melbourne, Australia; Boston Children's Hospital / Harvard Medical School, UNITED STATES

## Abstract

**Background:**

Clinicians and researchers require sound neurological tests to measure changes in neurological impairments necessary for clinical decision-making. Little evidence-based guidance exists for selecting and interpreting an appropriate, paediatric-specific lower limb neurological test aimed at the impairment level.

**Objective:**

To determine the clinimetric evidence underpinning neurological impairment tests currently used in paediatric rehabilitation to evaluate muscle strength, tactile sensitivity, and deep tendon reflexes of the lower limb in children and young people with a neurological condition.

**Methods:**

Thirteen databases were systematically searched in two phases, from the date of database inception to 16 February 2017. Lower limb neurological impairment tests were first identified which evaluated muscle strength, tactile sensitivity or deep tendon reflexes in children or young people under 18 years of age with a neurological condition. Papers containing clinimetric evidence of these tests were then identified. The methodological quality of each paper was critically appraised using standardised tools and clinimetric evidence synthesised for each test.

**Results:**

Thirteen papers were identified, which provided clinimetric evidence on six neurological tests. Muscle strength tests had the greatest volume of clinimetric evidence, however this evidence focused on reliability. Studies were variable in quality with inconsistent results. Clinimetric evidence for tactile sensitivity impairment tests was conflicting and difficult to extrapolate. No clinimetric evidence was found for impairment tests of deep tendon reflexes.

**Conclusions:**

Limited high-quality clinimetric evidence exists for lower limb neurological impairment tests in children and young people with a neurological condition. Results of currently used neurological tests, therefore, should be interpreted with caution. Robust clinimetric evidence on these tests is required for clinicians and researchers to effectively select and evaluate rehabilitation interventions.

## Introduction

Paediatric neurological examinations are a fundamental component for planning and adjusting rehabilitation interventions, monitoring the course of a neurological condition, and evaluating the effectiveness of an intervention, [1–3] in both clinical and research settings. Neurological examinations consist of a battery of neurological tests spanning multiple constructs of the International Classification of Function, Disability and Health: Children and Youth version (ICF-CY) framework. [4] Neurological impairment tests include those that evaluate muscle strength, tactile sensitivity, and deep tendon reflexes. These tests are frequently used to evaluate a child’s neural integrity [3,5,6] at the body functions and structures level of the ICF-CY [4] and may be used to aid in the selection of other tests including those in activity and participation domains. [3,5]

Clinicians may structure their physical assessment with an initial screen of neurological integrity in conjunction with the history, or subjective examination, providing information on activity limitations or participation restrictions from the child, young person or parent. Due to the increasing numbers of neurological impairment tests available, [3,6–11] selection of best available tests becomes difficult. There is no known “gold standard” for a neurological examination to aid in selecting tests [11–13] and/or “gold standards” for individual neurological impairment tests require expensive equipment that lack the clinical utility for daily practice, [14] such as the use of the isokinetic dynamometer for measuring muscle strength. Clinimetrics is a term that describes the psychometric properties of a test (reliability, validity, and responsiveness to change over time) and the test’s clinical utility (the clinical usefulness of the test) (Table 1). [7,10,14,15] Few paediatric tests have undergone comprehensive clinimetric evaluation. [5,12–15] Without specific clinimetric evidence, recommendations and clinical guidelines for using existing tests cannot be developed for use in children with a neurological condition.

**Table 1:** Clinimetric definitions for a lower limb neurological impairment test.

Domain	Measurement property	Definition
Reliability		The extent to which repeated scores for a neurological test in a stable child are the same (consistent) [8,10] measuring the proportion of variability that is due to “true”^a^ differences and “free” from measurement error. [8]
	Test-retest^b^	Degree to which an individual achieves the same result on a repeated test(s) without involvement from a health practitioner. [16]
	Inter-rater	Degree to which different health practitioners achieve the same result on the same occasion of testing [8]
	Intra-rater	Degree to which the same health practitioner achieves the same result on different occasions of testing in a stable child [8]
Validity		Degree in which a neurological test measures what it intends to measure [8]
	Face validity	Degree in which the neurological test appears to reflect the items required to measure the intended construct [8]
	Content validity	Degree to which the domain, muscle strength, tactile sensitivity or deep tendon reflexes, is comprehensively sampled by the items within the test.
	Internal consistency	Degree to which items are correlated, thus measuring the same construct. [7]
	Construct validity	Degree in which scores from one test relate to another in a manner that is consistent with a theoretically derived hypothesis. [7,8]
	Criterion validity^c^	Degree in which scores of a neurological test relate to a gold standard, if one exists. [7,8]
Responsiveness		Ability to of a neurological test to detect change over time in the construct being measured, also described in literature as “sensitivity to change” [8]
Clinical Utility		Multi-dimensional concept for use of a test in clinical practice [14]
	Appropriate	Evidence of test effectiveness for clinical decision-making or relevance within the clinical setting with minimal impact on existing management of child. [14]
	Accessible	Low cost resources for the neurological test, (e.g. equipment) [14]. Neurological test easily procured, including availability and supply of the test, and the quality of test materials. [14]
	Practicable	Complete and working administration and scoring instructions, practicable, including suitability for children under 18 years of age and for use in the clinical practice. [14] And whether any training or prior knowledge is required for the tester.
	Acceptable	Acceptability of the test to clinicians, children and families (utility vs burden), including ethical and psychological factors [14]

^a^ Mokkink et al. 2010 [8] explains that “the word ‘true’ must be seen in the context of the classical test theory, which states that any observation is composed of two components–a true score and error associated with the observation. ‘True’ is the average score that would be obtained if the scale were given an infinite number of times. It refers only to the consistency of the score and not to its accuracy”

^b^ Test-retest reliability is reserved for tests repeated on two or more occasions without a direct physical measure by a health practitioner. e.g. A questionnaire.

^c^ Criterion validity is the highest level of validity, however there is no gold standard for a neurological impairment test

Although adult neurological tests have frequently been modified for use in paediatric populations, the clinimetric properties of adult tests are not inherently transferrable to children and young people. [13,17] Adult tests tend to be modified for use in paediatric populations without a standardised protocol, [3,18] making it difficult to interpret the findings of these modified tests. Standardised protocols that increase a child’s comprehension and confidence to complete a task in a distraction-free environment are essential in reducing random errors, [16] particularly as a child grows and develops.

Clinimetric properties of tests for typically developing children are also not necessarily transferable to children with a lower limb neurological condition. [3,13,17–19] For example, children with neurological conditions may have intellectual disabilities that influence the child’s comprehension of the test requirements, and therefore their performance. Physical disabilities that also may influence a neurological test protocol and results include, but are not limited to, the presence of muscle contractures, spasticity or variations in tone, and previous orthopaedic surgery.

There is little evidence-based guidance on how to assist clinicians and researchers select and interpret an appropriate, paediatric-specific lower limb neurological test for children and young people with a neurological disorder. [12,20,21] While clinimetric evidence of activity and participation measures in children and young people with neurological diagnoses have been identified, [22] evidence of impairment measures remains limited. A recent systematic review found no conclusive clinimetric evidence to support the use of handheld dynamometry to measure muscle strength in children and young people with cerebral palsy, due to the poor methodological quality of primary papers. [23] Other systematic reviews have identified the lack of high quality clinimetric evidence for upper limb tests in children and young people with a neurological condition. [1,9] The clinimetric evidence for other lower limb neurological impairment tests for children and young people with cerebral palsy and other neurological conditions remains unknown. Therefore, the aims of this study were to:

- Identify neurological impairment tests currently used to evaluate the lower limb neural integrity of muscle strength, tactile sensitivity, and deep tendon reflexes in children and young people with a neurological condition
- Identify clinimetric evidence for neurological impairment tests used in children and young people with a wide range of neurological conditions
- Critically appraise and synthesise the clinimetric evidence underpinning the lower limb neurological tests
- Make recommendations regarding their use in clinical practice and research settings.

## Method

This study was undertaken in two phases based on the works by Bialocerkowski and colleagues. [21,24] The first phase systematically identified lower limb neurological tests measuring muscle strength, tactile sensitivity or deep tendon reflexes, in children and young people. [25] The second phase systematically identified studies evaluating the clinimetric properties of these neurological tests specific to children and young people with a neurological condition.

### Phase 1: Identification of neurological tests

Search terms, identifying lower limb neurological impairment tests for children (aged 2–18 years) with a neurological condition, were generated from previous search strategies. [26,27] Medical subject headings (MeSH) for ‘Lower Extremity’ AND (‘Neurological Examination’ OR ‘Physical Examination’) AND (‘Sensation’ OR ‘Reflex’ OR ‘Muscle Strength’) AND (‘Child’ OR ‘Adolescence’ OR ‘Child, Preschool’) were expanded to select relevant subcategories where possible. The search was simplified to ‘Sensation’, as results for ‘Touch Sense’ (the MeSH term for tactile sensitivity) were included within the broader search filter of ‘Sensation’. Neurological diagnoses were not individually searched in Phase 1 as not to limit neurological impairment tests to the number or type of diagnoses. Phase 1 therefore developed an extensive list of paediatric neurological impairment tests that could be used in Phase 2 of this study. A neurological condition included conditions classified under the International Statistical Classification of Diseases and Related Health Problems (ICD-10) codes. S1 Table displays the search strategy used for CINAHL. Thirteen health-related databases were systematically searched from January 1985 to 16 February 2017: CINAHL, Cochrane Library, EMBASE, Health Reference Center, Joanna Briggs Institute, Medline, PEDro, ProQuest Central, ProQuest Dissertations and Theses, ScienceDirect, Scopus, TRIP Database, Web of Science. Grey literature, including conference proceedings, theses and dissertations were included within database searches with no language limitations.

### Study selection

Duplicates were removed from identified papers, before two researchers (RC and BH) independently evaluated them for the following inclusion criteria:

1. Paediatric participants had an average age greater than two years and less than 18 years, as the focus of the paper was on children and young people less than 18 years. [25,28–30]
2. Participants with a neurological condition affecting the lower limb. These conditions included diseases of the nervous system, musculoskeletal system and connective tissue, injuries to the head or unspecified part of trunk and certain other consequences of external causes, certain conditions originating in the perinatal period, congenital malformations, deformations and chromosomal abnormalities that effect the central or peripheral nervous system including the spinal cord, peripheral nerves, nerve roots, autonomic nervous system and muscles. [2]
3. Papers reported using a neurological impairment test that measured or evaluated muscle strength (b730), and/or tactile sensitivity (b270), and/or deep tendon reflexes (b750) at the “body functions and structures” level of the ICF-CY. [4]
4. Neurological impairment tests were suitable for use within the clinical setting, using equipment that was typically available, inexpensive and portable. [15,31]
5. Quantitative studies with a level of evidence rated I-IV [32] (including systematic reviews (I), randomised controlled studies (RCTs)(II) and, pseudo-RCTs (III), comparative studies (III2,3), and case series with pre/post studies (IV))
6. Full text or abstract papers published in a peer-reviewed journal, as listed in Ulrichsweb. [32]
7. Published in the English language between 1985 to February 2017, as papers published after the mid-1980s were considered to coincide with a period for the use of evidence-based practice (EBP) to optimise clinical care. [33]

Papers were excluded if:

1. The average age for participants could not be determined or the average age of participants was younger than 2 years of age or older than 18 years of age.
2. Participants were diagnosed with conditions limited to metabolic, orthopaedic or cardiovascular conditions (including, but not limited to systemic connective tissue disorders and other osteopathies, episodic and paroxysmal disorders and inflammatory diseases of the central nervous system).
3. Neurological tests were classified as activity or participation measures, as these measures represented a different ICF-CY construct. [4]
4. Papers reported only spasticity or primitive reflexes, as these were not the focus of this study. [28–30]
5. Neurological impairment tests with a low level of clinical utility due to expense or limited transportability of equipment (e.g. isokinetic dynamometer) or the specialised diagnostic nature of testing (e.g. electromyography or nerve conduction studies). [14]
6. Papers were editorials or opinion pieces, as they are not quantitative studies. [34]

If eligibility was unclear, the two researchers (RC and BH) undertook a review of the full text article. A third reviewer (AB) was consulted to reach consensus in cases of continued disagreement. Included papers were reviewed in full text and the names of all relevant neurological tests were extracted by the same two researchers (RC and BH) and compared for agreement. If required, the third reviewer (AB) determined consensus.

### Phase 2: Identification of clinimetric properties of neurological tests

Neurological impairment tests identified in Phase 1 were systematically searched for their clinimetric properties from their date of inception to 16 February 2017 using four health databases, CINAHL, EMBASE, Medline, Scopus. [24] By translating the validated Terwee, Jansma, Riphagen, et al. [35] protocol for each specific database (S2 Table), the search strategy involved combining:

- a neurological test search, to identify measures of muscle strength, or tactile sensitivity, or deep tendon reflexes limited to the lower limb;
- a population search, including paediatric participants aged less than 18 years;
- a neurological test search, derived from the neurological impairment test names identified in Phase 1 and;
- filtering for measurement properties, as outlined by Terwee, et al. [35]

Papers were included if:

1. all paediatric participants were aged less than 18 years, as clinimetric properties are population specific. [17]
2. participants had a neurological condition affecting the lower limb. Neurological conditions were defined using the International Classification of Diseases (ICD-10) as per Phase 1.
3. papers contained clinimetric evidence on a lower limb neurological impairment test that evaluated muscle strength, tactile sensitivity and/or deep tendon reflexes in the lower limb as per the ICF-CY framework outlined in Phase 1.
4. quantitative studies with a level of evidence rated II-IV [34] (including randomised controlled studies (RCTs)(II) and, pseudo-RCTs (III), comparative studies (III2,3), and case series with pre/post studies (IV))
5. papers were published in full text in the English language and peer reviewed.

Consensus between two individual reviewers (RC and BH) was reached using the same method as Phase 1. Papers that contained additional evidence outside the scope of this paper were included only if data could be extrapolated that met the inclusion criteria. Systematic reviews (level I evidence) identified in this process were searched for primary papers that met the inclusion criteria through secondary searching. Additional primary papers that met the inclusion criteria were identified through secondary searching by hand through the reference lists of included papers and identified systematic reviews.

### Quality assessment

The methodological quality of the included clinimetric papers was evaluated independently by two reviewers (RC and BH) using two critical appraisal tools: Brink and Louw critical appraisal tool [6] and the COnsensus-based Standards for the selection of health Measurement INstruments (COSMIN). [8] These critical appraisal tools [6,8] have previously been used in a number of published systematic reviews on health-related outcome measures [21,36–39] to evaluate the aspects of the quality of psychometric evidence. Brink and Louw’s [6] tool assessed the impact of 13 items on the overall quality of the primary paper’s method, without calculating a composite score [6,21]. For each included primary paper, the percentages of “yes” responses for applicable items [6] was calculated by dividing the number of “yes” responses by the number of applicable items and converted into a percentage. [36,40] This provided an arbitrary evaluation of the overall methodological quality of each paper. Due to its wide use in health-related research the COSMIN was used to grade the methodological quality of included papers. [1,20,22,31,41] The COSMIN uses weighted items based on overall importance and a ‘worst score counts’ method. [42] Consensus for each item was gained through discussion and a third researcher (AB) was consulted if required. Kappa coefficients and 95% confidence intervals (CI) were calculated to assess the inter-reviewer reliability of the item response.

### Data extraction

Additional data were extracted from each study, including the name of the authors, date of publication, name of the neurological test, type of clinimetric property evaluated, participant characteristics, rater characteristics, measurement characteristics, results of the clinimetric evaluation and information on the clinical utility of the test. Clinical utility was described based on information contained within the included papers on the portability, cost, and feasibility of using the equipment on children and young people with a neurological condition in a clinical setting. [14]

### Best evidence synthesis

Evidence on each clinimetric property for each neurological test within primary papers was narratively synthesised and interpreted in combination with the methodological quality of the primary paper. Reliability correlation coefficients from the primary papers were interpreted using guidelines from Katz et al., [43] low = <0.40, moderate = 0.40–0.59, moderately high = 0.60–0.79 and very high = >0.80. The level of evidence for each neurological test was determined using guidelines from Terwee et al. [7] and Dobson et al., [31] which combined the quality of the paper for each neurological test with the consistency of the clinimetric evidence for that test (Table 2). [20,31,44]

**Table 2:** Levels of evidence synthesis for methodological quality of paper and consistency of clinimetric evidence of measurement property.^a^.

Level	Rating	Criteria
Strong evidence	+++ or—-	Consistent findings in multiple studies of good methodological quality OR in one study of excellent methodological quality
Moderate evidence	++ or —	Consistent findings in multiple studies of fair methodological quality OR in one study of good methodological quality
Limited evidence	+ or -	One study of fair methodological quality
Conflicting evidence	±	Conflicting findings
Unknown evidence	?	Only studies of poor methodological quality

+ = positive rating,— = negative rating, ± = conflicting rating,? = indeterminate rating

^a^Adapted from Terwee et al., [7] Dobson et al. [31] and Dekkers et al. [20]

## Results

### Search output

**Fig 1:**
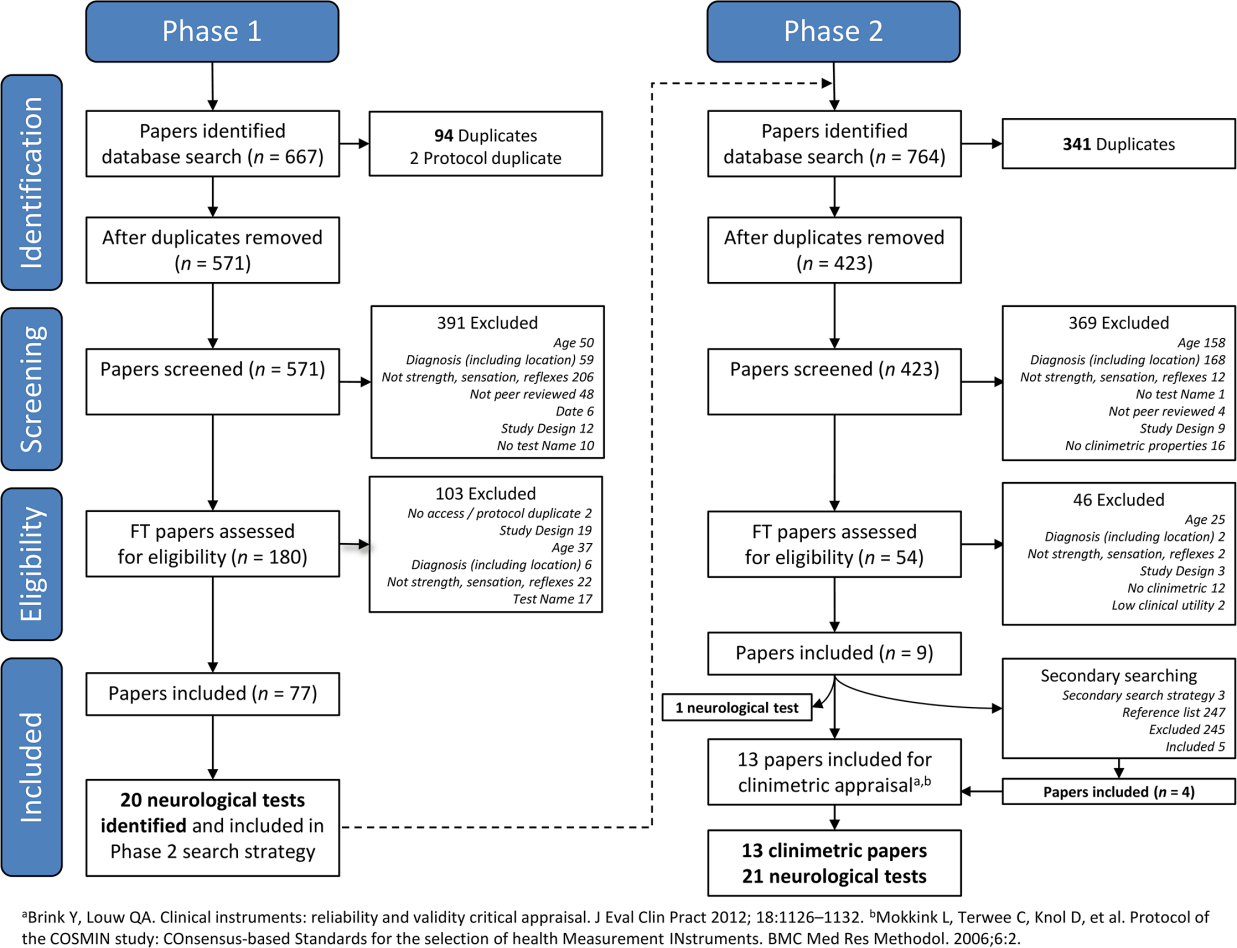
PRISMA flow diagram of systematic search strategy used to identify clinimetric papers.

The Phase 1 search strategy identified 77 papers that met the inclusion criteria. Thirteen papers [45–57] met the Phase 2 selection criteria with clinimetric evidence of a neurological test (Fig 1). Twenty-one lower limb neurological tests were identified in total: ten evaluated muscle strength, six tactile sensitivity, one deep tendon reflexes, and four evaluated a combination of these constructs (S3 Table).

### Methodological quality

There was high agreement (kappa = .98, 95% CI 0.96–1.01) between reviewers when scoring the methodological quality of included papers. The most prevalent quality limitations identified using the Brink and Louw critical appraisal tool [6] and COSMIN [8] were a lack of rater blinding, a lack in variation of examination order, not reporting the stability of the child’s condition and small samples of less than 30, with the exception of Florence et al. [56] (Table 3). Most primary papers rated “yes” for greater than 60% of Brink and Louw’s criteria (n = 11/13), [45–47,49–53,56] with a range from 38% “yes” statements [55] to 88% “yes” statements. [54] Thirteen [45–56] primary papers were rated as poor quality using the COSMIN criteria [8] (Table 4). This was mainly because primary papers had a sample size of less than 30 participants.

**Table 3:** Brink and Louw^a^ critical appraisal summary of the methodological quality of the clinimetric papers. (*n* = 15).

Authors	Neurological Test	Diagnosis	Item 1	Item 2	Item 3	Item 4	Item 5	Item 6	Item 7	Item 8	Item 9	Item 10	Item 11	Item 12	Item 13	% “Yes”
Berry [46]	HHD	CP	Y	Y	N/A	N/A	N	N	N/A	N	N/A	Y	N/A	Y	Y	**63**
Burns [45]	CMTPedS	CMT	Y	Y	N/A	N	N/A	N	N/A	Y	N/A	Y	N/A	Y	Y	**75**
Crompton [47]	HHD	CP	Y	N	N/A	N/A	Y	Y	N/A	N	N/A	Y	N/A	N	Y	**63**
Effgen [48]	HHD	SB	Y	N	N/A	N	Y	N	N/A	N	N/A	Y	N/A	Y	Y	56
Escolar [55]	MMT, RQMS	DMD	Y	Y	N/A	N	N/A	N	N/A	N	N/A	N	N/A	Y	Y	38
Florence [56]	MMT	DMD	Y	Y	N/A	N/A	Y	N	N/A	N	N/A	N	N/A	Y	Y	**63**
Mahony [49]	HHD, MMT	SB	Y	Y	N/A	N	N/A	Y	N/A	Y	N/A	N	N/A	Y	N	**63**
Mulcahey [57]	ASIA Scale	SCI	Y	N	N/A	N/A	N	N	N/A	Y	N/A	N	N/A	Y	Y	50
Stuberg [50]	HHD	DMD	Y	N	N/A	N/A	N	N	N/A	Y	N/A	Y	N/A	Y	Y	**63**
Taylor [51]	HHD	CP	Y	Y	N/A	N/A	N	N	N/A	N	N/A	Y	N/A	Y	Y	**63**
Van Vulpen [52]	HHD, SHR	CP	Y	Y	N/A	N/A	Y	N	N/A	N	N/A	Y	N/A	Y	Y	**75**
Verschuren [53]	HHD	CP	Y	Y	N/A	N	N/A	N	N/A	Y	N/A	Y	N/A	Y	Y	**75**
Williemse [54]	HHD	CP	Y	Y	N/A	N/A	Y	N	N/A	Y	N/A	Y	N/A	Y	Y	**88**

CMTPedS, Charcot-Marie-Tooth Pediatric Scale; HHD, Hand-held dynamometer; MMT, Manual Muscle Test; RQMS, Richmond Quantitative Measurement System; SHR, Standing Heel Rise Test. CP, Cerebral Palsy; CMT, Charcot-Marie-Tooth; SB, Spina Bifida; DMD, Duchenne’s Muscular Dystrophy; Y = ‘Yes’, N = ‘No’, N/A = not applicable. Item 1: If human subjects were used, did the authors give a detailed description of the sample of subjects used to perform the (index) test? Item 2: Did the author’s clarify the qualification, or competence of the rater(s) who performed the (index) test? Item 3: Was the reference standard explained? Item 4: If inter-rater reliability was tested, were raters blinded to the findings of other raters? Item 5: If intrarater reliability was tested, were raters blinded to their own prior findings of the test under evaluation? Item 6: Was the order of examination varied? Item 7: If human participants were used, was the time period between the reference standard and the index test short enough to be reasonably sure that the target condition did not change between the two tests? Item 8: Was the stability (or theoretical stability) of the variable being measured taken into account when determining the suitability of the time interval between repeated measures? Item 9: Was the reference standard independent to the index test? Item 10: Was the execution of the (index) test described in sufficient detail to permit replication of the test? Item 11: Was the execution of the reference standard described in sufficient detail to permit its replication? Item 12: Were withdrawals from the study explained? Item 13: Were the statistical methods appropriate for the purpose of the study?

% “Yes” Are calculated from the number of “yes” responses to applicable items only, items > 60% are shown in bold.

^a^Adapted from Brink and Louw et al. [6]

**Table 4:** COSMIN^a^ reliability critical appraisal summary of the methodological quality of the clinimetric papers. (*n* = 15).

Authors	Neurological Test	Diagnosis	Item 1	Item 2	Item 3	Item 4	Item 5	Item 6	Item 7	Item 8	Item 9	Item 10	Item 11	Item 12	Item 13	Item 14	COSMIN Grade
Berry [46]	HHD	CP	1	1	4	1	2	1	2	1	1	3	1	N/A	N/A	N/A	Poor
Burns [45]	CMTPedS	CMT	2	2	4	1	2	1	2	1	2	3	1	N/A	N/A	N/A	Poor
Crompton [47]	HHD	CP	2	1	4	1	1	1	3	1	1	1	1	N/A	N/A	N/A	Poor
Effgen [48]	HHD	SB	1	2	4	1	1	1	3	3	2	3	1	N/A	N/A	N/A	Poor
Escolar [55]	MMT, RQMS	DMD	2	2	4	1	2	1	3	1	1	4	2	N/A	N/A	2	Poor
Florence [56]	MMT	DMD	2	2	1	1	1	1	4	4	1	4	1	N/A	1	2	Poor
Mahony [49]	HHD, MMT	SB	1	2	4	1	1	1	3	3	1	4	4	N/A	N/A	1	Poor
Mulcahey [57]	ASIA Scale	SCI	2	3	3	1	2	1	1	1	2	4	2	N/A	N/A	N/A	Poor
Stuberg [50]	HHD	DMD	1	1	4	1	2	1	2	1	1	4	3	N/A	N/A	N/A	Poor
Taylor [51]	HHD	CP	2	3	4	1	2	1	2	1	2	3	1	N/A	N/A	N/A	Poor
Van Vulpen [52]	HHD, SHR	CP	1	1	4	1	1	1	2	1	1	1	1	N/A	N/A	N/A	Poor
Verschuren [53]	HHD	CP	1	1	4	1	2	1	2	1	1	3	2	N/A	N/A	N/A	Poor
Williemse [54]	HHD	CP	1	1	4	1	1	1	1	1	1	1	1	N/A	N/A	N/A	Poor

CMTPedS, Charcot-Marie-Tooth Pediatric Scale; HHD, Hand-held dynamometer; MMT, Manual Muscle Test; RQMS, Richmond Quantitative Measurement System; SHR, Standing Heel Rise Test. CP, Cerebral Palsy; CMT, Charcot-Marie-Tooth; HHD, Hand held dynamometer; SB, Spina Bifida; DMD, Duchenne’s Muscular Dystrophy; COSMIN Grades: 1 = excellent, 2 = good, 3 = fair, 4 = poor, N/A = non-applicable. Item 1: Was the percentage of missing items given? Item 2: Was there a description of how missing items were handled? Item 3: Was the sample size included in the analysis adequate? Item 4: Were at least two measurements available? Item 5: Were the administrations independent? Item 6: Was the time interval stated? Item 7: Were patients stable in the interim period on the construct to be measured? Item 8: Was the time interval appropriate? Item 9: Were the test conditions similar for both measurements? e.g., type of administration, environment, and instructions Item 10: Were there any important flaws in the design or methods of the study? Item 11: For continuous scores: Was an intraclass correlation coefficient (ICC) calculated? Item 12: For dichotomous/nominal/ordinal scores: Was kappa calculated? Item 13: For ordinal scores: Was a weighted kappa calculated? Item 14: For ordinal scores: Was the weighting scheme described? e.g. linear, quadratic

^a^COSMIN methodological quality using Box B on reliability adapted from Mokkink 2010 et al. [8] as there was no evidence on validity or responsiveness

### Neurological tests and their clinimetric properties

The 13 primary papers provided clinimetric evidence for six neurological tests: American Spinal Impairment Association) ASIA impairment scale, [57] Charcot-Marie-Tooth Pediatric Scale, [45] Handheld Dynamometry (HHD), [46–54] Manual Muscle Test (MMT), [49,55,56] Richmond Quantitative Measurement System, [55] and Standing Heel Rise. [52] All tests evaluated lower limb muscle strength. The ASIA impairment scale and Charcot-Marie-Tooth Pediatric Scale evaluated tactile sensitivity and muscle strength in combination with other upper and lower limb tests to form a composite score. [45,57] No studies evaluated the clinimetric properties of lower limb deep tendon reflexes. All identified clinimetric evidence focused on the reliability of the test. No primary papers evaluated validity, responsiveness or clinical utility of the test. Reliability evidence was generated from participants with a range of neurological conditions, including six papers examining neurological tests in children and young people less than 18 years of age with cerebral palsy, [46,47,51–54] one with Charcot-Marie-Tooth, [45] one with spinal cord injury, [57] three with Duchenne’s muscular dystrophy [50,55,56] and two papers with children with spina bifida. [48,49] Children varied in age from 3 years 8 months [52] to 18 years. [51] Physiotherapists with over six years of experience performed neurological tests in all primary papers (S4 Table).

### Clinimetric evidence

#### Hand held dynamometry

**Fig 2:**
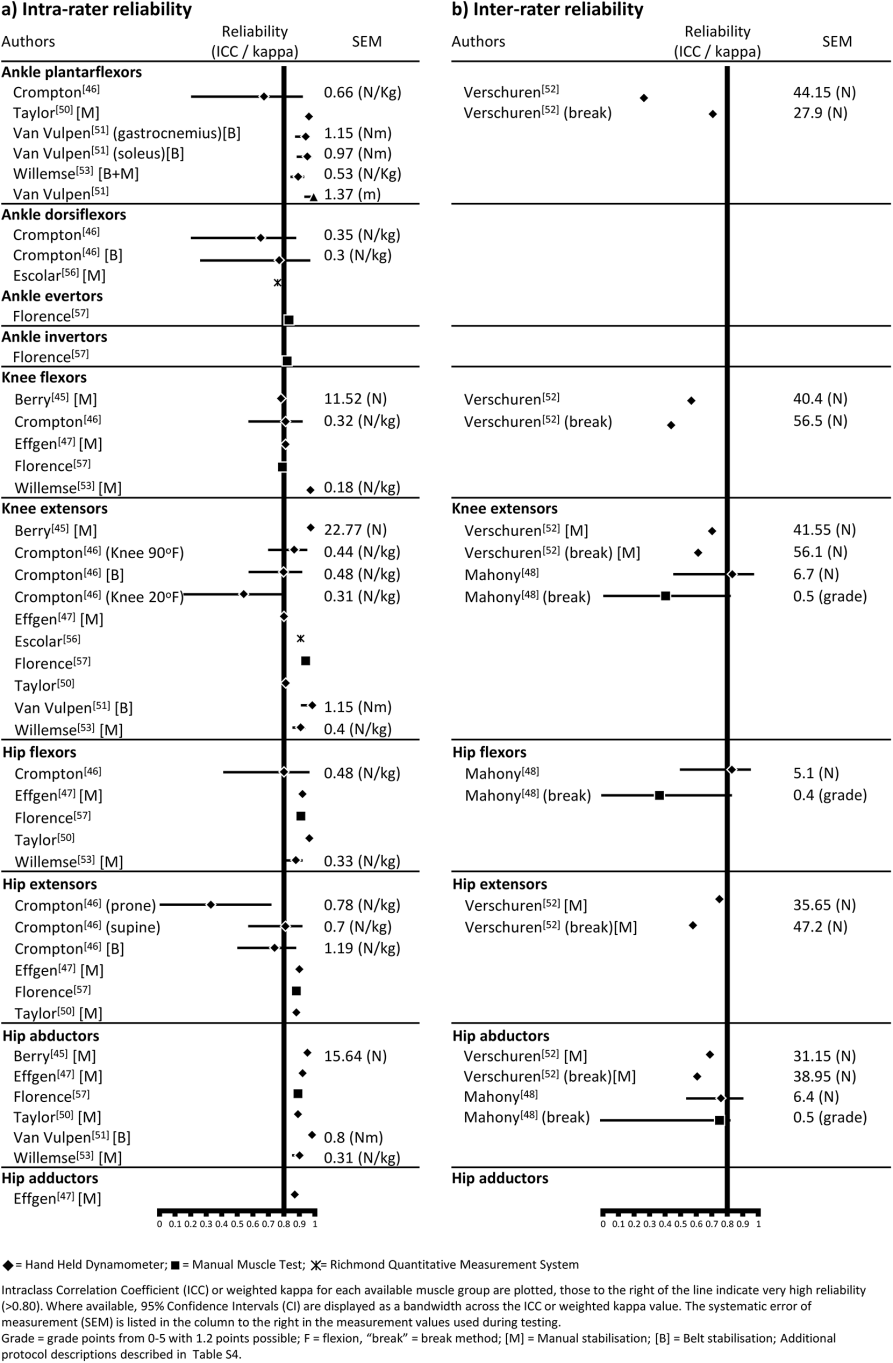
Forest plot of intra-rater and inter-rater reliability ICC with 95% CI and systematic error of measurement (SEM), where available, for muscle strength tests using different protocols.

Hand held dynamometry had the largest body of evidence, with nine papers [46–54] reporting evidence of reliability. (Table 5) The majority of clinimetric evidence was identified in six papers [46,47,51–54] for children with cerebral palsy. The remaining papers had evidence for children with spina bifida [48,49] and Duchenne’s muscular dystrophy. [50] Eight of the primary papers [46,47,49–54] evaluating hand held dynamometry had greater than 60% of “yes” items on methodological quality with a range of 56%-88% using the Brink and Louw [6] criteria (Table 3). Yet, all papers [46,47,49–54] had poor methodological quality according to the COSMIN checklist [8] due to the sample sizes being considered small (Table 4). Most papers reported moderately high to very high intra-rater reliability with ICCs ranging from 0.70 to 0.99. [46–48,51,52,54] Conversely, Crompton et al. [47] reported low reliability with ICCs as low as 0.26. Inter-rater reliability was more variable than intra-rater reliability across all papers with ICCs ranging from -0.04 to 0.97 [53,55] (Table 5). The “make” measurement method (Table 6) to evaluate strength had the largest body of evidence [45–54] (Table 5) with higher ICC values compared to the “break” method [53] (Fig 2). Manual or belt stabilisation of the proximal limb consistently had slightly higher intrarater reliability (Fig 2) compared with no stabilisation, particularly for the hip and knee extensors. [47] Variable confidence intervals were reported in the four papers [47,49,52,54] that calculated 95% CI (Fig 2). Protocols were not standardised across studies with different muscle groups tested, multiple body positions adopted (e.g. supine or sitting), variable placement of the dynamometer, different equipment used with numerous units of measurement, and disparate lengths of time between repeated tests (S4 Table). HHD had high portability, yet requires specialised equipment, reducing its clinical utility. [14] The cost of equipment was not reported and a requirement for additional training was inconsistently described. [46–54]

**Table 5:** The clinimetric properties of neurological tests for children and young people with a neurological condition.

Neurological test	Population	Intra-rater reliability	Inter-rater reliability	SEM	MDC
ASIA impairment scale (Incl. MMT, pinprick, light touch) [57]	SCI (*n = 48*) [57]	ICC = 0.71–0.98 [57]		Not reported	Not reported
Charcot-Marie-Tooth Pediatric Scale (Incl. HHD) (*n* = 1) [45]	CMT (*n* = 8) [45]		ICC = 0.95 (range unknown) [45]	Not reported	Not reported
Hand held dynamometer (*n* = 9) [46–54]	CP (*n* = 107) [46,47,51–54]	ICC = 0.71–0.97 [46]		6.72–19.88 (N) [46]	Not reported
		ICC = 0.26–0.91 [47]		0.20–1.30 (N/kg) [47]	Not reported
		ICC = 0.81–0.98 [51]		Not reported	Not reported
		ICC = 0.81–0.99 [52]		0.6–1.56 (Nm) [52]	3.87–3.88 (Nm) [52]
			Make: ICC = -0.04–0.82 [53]	30.6–52.7 (N) [53]	Not reported
			Break: ICC = 0.42–0.73 [54]	27.9–58.9 (N) [55]	Not reported
		ICC = 0.87–0.97 [54]		0.18–0.53 (N/kg) [54]	0.51–2.27 [54]
	SB (*n* = 20) [48,49]	ICC = 0.73–0.96 [48]	Not reported^a^	Not reported	Not reported
			ICC = 0.76–0.83 [49]	5.10–6.70 (N) [49]	11.88–15.41 (90%) [49]
	DMD (*n* = 14) [50]	r = 0.83–0.99 [50]		Not reported	Not reported
Manual muscle test (*n* = 3) [49,55,56]	SB (*n* = 20) [49]		ICC = 0.37–0.75 [49]	0.40–0.50 (0–5 ordinal scale) [49]	0.85–1.27 (90%) [49]
	DMD (*n* = 109*)* [55,56]	κ = 0.71–0.93 [56]		Not reported	Not reported
	BMD (*n* = 2) [55]		Unable to report^b^		
	LGMD (*n* = 3) [55]				
Standing Heel Rise (*n* = 1) [52]	CP (*n* = 20) [52]	ICC = 0.91–0.99 [52]		0.45–1.38 (m) [52]	1.7–6.1 (Nm) [52]
Richmond Quantitative Measurement System (*n* = 1) [55]	DMD (*n* = 7) [55]		ICC = 0.56–0.97 [55]	Not reported	Not reported
	BMD (*n* = 2) [55]				
	LGMD (*n* = 3) [55]				

ICC, intraclass correlation coefficient; SEM, Standard Error of Measurement; MDC, Minimal Detectible Change; r, Pearson product moment correlation coefficient; κ, weighted kappa; HHD, Hand held dynamometer; CMT, Charcot-Marie Tooth; CP, Cerebral Palsy; SB, Spina Bifida; DMD, Duchene’s Muscular Dystrophy; BMD, Becker’s Muscular Dystrophy; LGMD, Limb Girth Muscular Dystrophy

^a^Effgen et al [48] assessed inter-rater reliability, however no data was reported therefore could not be discussed.

^b^Escolar et al. [55] calculated inter-rater reliability, however lower limb data could not be extrapolated.

**Table 6:** Muscle groups with protocols for testing lower limb strength in children and young people with a neurological condition.

Muscle groups (number of papers, n =)		Body and Limb position	Diagnosis	Equipment	Equipment placement	Body part stabilised	Test type	Trial used
**Ankle Plantarflexors (n = 7) [45,47,51–54,56]**								
	Sitting [45]	Knee extended 0^o^, hips flexed [45]	CP [45]	HHD, Citec [45]	plantar surface of foot, proximal to metatarsal heads [45]	lower leg, proximal to ankle joint [45]	Make [45]	Mean [45]
	Not reported [56]	Not reported [56]	DMD [56]	MMT (modified MRC scale 0–5 with +/- for grades 3–5) [56]	plantar surface of metatarsal heads^a^ [56]	Nil [56]	Make [56]	Not reported [56]
	Supine [47,51–54]	Knee extended 0^o^, foot plantargrade [47]	CP [47]	HHD, Nicholas Manual muscle tester [47]	plantar surface of metatarsal heads [47]	nil [47]	Make [47]	Peak [47]
		hips 45^o^, knees extended [52,53]	CP [52]	HHD, MicroFET, Biometrics [52]	metatarsal heads [52]	pelvis with belt, manually on lower limb [52]	Make [52]	Peak [52]
			CP [53]	HHD, Citec [53]	dorsum of foot at level of metatarsal heads^b^ [53]	lower leg [53]	Make [53] Break [53]	Peak [53]
		Knee flexed 90^o^, hip 90^o^, ankle neutral [51,52,54]	CP [51]	HHD, Nicholas Manual muscle tester [51]	metatarsal heads [51]	lower leg [51]	Make [51]	Mean [51]
			CP [52,54]	HHD, MicroFET, Biometrics [52,54]	plantar surface of metatarsal heads [52,54]	pelvis with belt, manually on lower limb [52,54]	Make [52,54]	Peak [52], All trials [54]
	Standing [52]	Hip and knee extended [52]	CP [52]	SHR [52]	nil [52]	nil [52]	Dynamic [52]	Not reported [52]
		**Ankle Dorsiflexors (n = 4) [45,47,55,56]**					
	Sitting [45,55]	Knee extended 0^o^ [45]	CMT [45]	HHD, Citec [45]	dorsal surface of foot, proximal to metatarsal heads [45]	lower limb, proximal to ankle joint [45]	Make [45]	Mean [45]
		Not reported [55]	DMD [55]	RQMS strain gauge, Interface SM-50-12 [55]	Not reported [55]	back support [55]	Make [55]	Peak [55]
	Not reported [56]	Not reported [56]	DMD [56]	MMT (modified MRC scale 0–5 with +/- for grades 3–5) [56]	Dorsal surface of foot over 1^st^ metatarsal^a^ [56]	Nil [56]	Make [56]	Not reported [56]
	Supine [47]	Knee extended 0^o^ [47]	CP [47]	HHD, Nicholas Manual muscle tester [47]	dorsal surface of metatarsal heads [47]	nil [47]	Make [47]	Peak [47]
						thigh, using stabilising belt [47]	Make [47]	Peak [47]
		**Ankle Evertors (n = 1) [56]**						
	Not reported [56]	Not reported [56]	DMD [56]	MMT (modified MRC scale 0–5 with +/- for grades 3–5) [56]	lateral aspect of foot over 5th metatarsal^a^ [56]	nil [56]	Make [56]	Not reported [56]
		**Ankle Invertors (n = 1) [56]**						
	Not reported [56]	Not reported [56]	DMD [56]	MMT (modified MRC scale 0–5 with +/- for grades 3–5) [56]	medial aspect of foot over 1st metatarsal^a^ [56]	nil [56]	Make [56]	Not reported [56]
		**Knee Flexors (n = 8) [46–48,53–56,56]**						
	Sitting [46–48,53–56]	Knee and hip flexed 90^o^ [46–48,53–56]	CP [46]	HHD, Chatillion [46]	posterior calf, ~4cm proximal to malleoli [46]	thigh [46]	Make [46]	Peak [46]
			CP [47]	HHD, Nicholas Manual muscle tester [47]	proximal to bimalleolar line^c^ [47]	nil [47]	Make [47]	Peak [47]
			CP [54]	HHD, MicroFET, Biometrics [54]	posterior calf, 5 cm proximal to malleoli [54]	pelvis, thigh [54]	Make [54]	All trials [54]
			CP [53]	HHD, Citec [53]	posterior calf, 5cm proximal to lateral malleolus [53]	thigh [53]	Make [53] Break [53]	Peak [53]
			SB [48]	HHD, Spark [48]	posterior calf, proximal to malleoli^d^ [48]	Thigh [48]	Make [48]	Peak [48]
	Not reported [56]	Not reported [56]	DMD [56]	MMT (modified MRC scale 0–5 with +/- for grades 3–5) [56]	posterior leg, proximal to ankle^a^ [56]	nil [56]	Make [56]	Not reported [56]
		**Knee Extensors (n = 13) [46–56]**						
	Sitting [46–55]	Knee and hip 90^o^ [46–55]	CP [46]	HHD, Chatillion [46]	anterior calf, ~2 cm proximal to malleoli [46]	pelvis [46]	Make [46]	Peak [46]
			CP [47,51]	HHD, Nicholas Manual muscle tester [47,51]	anterior tibia, proximal to bimalleolar line [47]	nil [47]	Make [47]	Peak [47]
					anteriorly, 5 cm proximal to lateral malleolus [47,51]	pelvis in chair using a belt [47]	Make [47]	Peak [47]
						nil [51]	Make [51]	Mean [51]
			CP [53]	HHD, Citec 4 [53]	anteriorly, 5 cm proximal to lateral malleolus [53]	pelvis [53]	make [53] break [53]	Peak [53]
			SB, [48] DMD [50]	HHD, Spark [48,50]	anterior leg, proximal to ankle [48,50]	Thigh [48,50]	Make [48,50]	Peak [48]
			CP [52,54]	HHD, MicroFET, Biometrics [52,54]	anterior tibia, 5 cm proximal from bimalleolar line [52,54]	pelvis in chair with belt, lumbar stabilisation adjusted with back of chair [52]	Make [52]	
						pelvis, thigh [54]	Make [54]	All trials [54]
			SB [49]	HHD, PowerTrack II Commander, Each [49]	anteriorly, mid-way between apex of patella and talocrural joint [49]	nil [49]	Make [49]	Mean [49]
			SB [49]	MMT (0–5 scale with 1/2 points) [49]	Not reported [49]	Not reported [48]	Not reported [48]	Peak [48]
			DMD [55]	RQMS strain gauge, Interface SM-50-12 [55]	Not reported [55]	back support [55]	Make [55]	
		Knee flexed 20^o^ [47]	CP [47]	HHD, Nicholas Manual muscle tester [47]	anterior tibia, proximal to bimalleolar line [47]	nil [47]	Make [47]	Peak [47]
	Not reported [56]	Not reported [56]	DMD [56]	MMT (modified MRC scale 0–5 with +/- for grades 3–5) [56]	anterior tibia, proximal to bimalleolar line^a^ [56]	nil [56]	Make [56]	Not reported [56]
		**Hip Flexors (n = 6) [47–49,51,54,56]**						
	Sitting [47,51,54]	Hip flexed, off surface [54]	CP [54]	HHD, MicroFET, Biometrics [54]	anterior thigh, 3 cm proximal to patella [54]	Pelvis [54]	Make [54]	All trials [54]
		Hip flexed 90 [47]	CP [47]	HHD, Nicholas Manual muscle tester [47]	anterior thigh, proximal to knee above superior border of patella [47]	nil [47]	Make [47]	Peak [47]
		Hip 30^o^ [51]	CP [51]	HHD, Nicholas Manual muscle tester [51]	anterior thigh, distally [51]	nil [51]	Make [51]	Mean [51]
	Supine [48,49]	Not reported [49]	SB [49]	HHD, PowerTrack II Commander, Each [49]	mid-way between ASIS and base of patella [49]	nil [49]	Make [49]	Mean [49]
			SB [49]	MMT (0–5 scale with 1/2 points) [49]	mid-way between ASIS and base of patella [49]	nil [49]	Not reported [49]	Peak [49]
		Hip and knee flexed 90^o^ [48]	SB [48]	HHD, Spark [48]	anterior thigh, proximal to knee^d^ [48]	Trunk [48]	Make [48]	Peak [48]
	Not reported [56]	Not reported [56]	DMD [56]	MMT (modified MRC scale 0–5 with +/- for grades 3–5) [56]	anterior thigh, proximal to knee above superior border of patella^a^ [56]	nil [56]	Make [56]	Not reported [56]
		**Hip Extensors (n = 6) [47,48,50,51,53,56]**						
	Prone [47,51]	Knee extended 0^o^ and thigh extended off surface [51]	CP [51]	HHD, Nicholas Manual muscle tester [51]	posterior distal thigh [51]	Pelvis [51]	Make [51]	Mean [51]
		Knee flexed 90^o^, hip extended off surface [47]	CP [47]	HHD, Nicholas Manual muscle tester [47]	posterior thigh, proximal to popliteal crease [47]	nil [47]	Make [47]	Peak [47]
	Supine [47,48,50,53]	Hip and knee flexed 90o [47, 48, 50]	CP [47]	HHD, Nicholas Manual muscle tester [47]	posterior thigh, proximal to popliteal crease [47]	nil [47]	Make [47]	Peak [47]
					posterior thigh, proximal to popliteal crease [47]	pelvis to plinth using belt [47]	Make [47]	Peak [47]
			SB, [48] DMD [50]	HHD, Spark [48,50]	posterior thigh, proximal to knee [48,50]	Trunk [48,50]	Make [48,50]	Peak [48] Mean [50]
		hip 90 [53]	CP [53]	HHD, Citec [53]	anterior mid-thigh [53]	pelvis [53]	Make [53] Break [53]	Peak [53]
	Not reported [56]	Not reported [56]	DMD [56]	MMT (modified MRC scale 0–5 with +/- for grades 3–5) [56]	posterior leg, proximal to bimalleolar line^a^ [56]	nil [56]	Make [56]	Not reported [56]
		**Hip Abductors (n = 8) [46,48,49, 51–54,56]**						
	Supine [46,48,51–54]	Hip and knees extended 0^o^ [46,47]	CP [46]	HHD, Chatillion [46]	lateral femur, ~5cm proximal from femoral epicondyle [46]	support of contralateral pelvis [46]	Make [46]	Peak [46]
			SB [48]	HHD, Spark [48]	lateral thigh, proximal to knee^d^ [48]	Contralateral lower extremity [48]	Make [48]	Peak [48]
		Hips neutral, 0^o^ abduction/adduction [53]	CP [53]	HHD, Citec [53]	lateral mid-thigh [53]	pelvis [53]	Make [53] Break [53]	Peak [53]
		Hip 45, knee extended [52], Hip slightly flexed off surface [54]	CP [52,54]	HHD, MicroFET, Biometrics [52,54]	5 cm proximal from femoral epicondyle [52]	pelvis with belt, contralateral limb held in neutral manually [52]	Make [52]	Mean [52]
					lateral thigh, 5 cm proximal to knee joint [54]	pelvis [54]	Make [54]	All trials [54]
		Hip and knee flexed 45^o^ [51]	CP [51]	HHD, Nicholas Manual muscle tester [51]	lateral thigh, distally [51]	Pelvis [51]	Make [51]	Mean [51]
	Side lying [49]	Knee extended 0^o^ [49]	SB [48]	HHD, PowerTrack II Commander [49]	lateral leg, midway between ASIS and patella [49]	nil [49]	Make [49]	Mean [49]
			SB [49]	MMT (0–5 scale with 1/2 points) [49]	Not reported	Not reported	Not reported	Peak [49]
	Not reported [56]	Not reported [56]	DMD [56]	MMT (modified MRC scale 0–5 with +/- for grades 3–5) [56]	posterior leg, proximal to bimalleolar line^a^ [56]	nil [56]	Make [56]	Not reported [56]
		**Hip Adductors (n = 1) [47]**						
	Supine [48]	Hip and knees extended 0^o^ [48]	SB [48]	HHD, Spark [48]	medial thigh, proximal to knee^d^ [48]	Contralateral lower extremity [48]	Make [48]	Peak [48]

^a^Positions as per Florence et al.’s [56] reference to Medical Research Council of the United Kingdom. Aids to examination of the peripheral nervous system: Memorandum No 45. Palo Alto, Calif: Pedragon House; 1978.

^b^This placement is a direct quote from Verschuren et al’s [53] original article. The dorsum of foot is a likely error as the plantarflexor muscle group is in the posterior compartment.

^c^This placement is a direct quote from Crompton et al.’s [47] original article. Resistance to the tibia is likely posterior as the knee flexor muscle group is in the posterior compartment.

^d^ Protocol as per Effgen et al.’s [48] reference to Bohannon RW. Test-retest reliability of hand-held dynamometry during a single session of strength assessment. Phys Ther. 1986;66:206–209. CP, Cerebral Palsy; CMT, Charcot-Marie-Tooth; DMD, Duchenne’s muscular dystrophy; SB, Spina Bifida; NR, not reported; MRC, Medical Research Council; ASIS, Anterior superior iliac spine; MMT, Manual Muscle Test; HHD, Hand-held dynamometer; ICC, Intra-class Coefficient; SEM, Standard Error of Measurement/the mean

#### Manual muscle testing

There was a small body of evidence for assessing muscle strength with MMT (n = 3 papers) [49,55,56] for children and young people with Duchenne’s muscular dystrophy [55,56] and spina bifida. [49] The methodological quality of primary papers ranged from 38% [55] to 63% [55,56] of “yes” items using the Brink and Louw method (Table 3). [6] All papers using MMT [49,55,56] had a poor methodological quality according to the COSMIN checklist, [8] due to sample sizes being considered small ^[^^49^^,^^55^^]^ and a lack of reported patient stability between testing sessions (Table 4). [56] MMT intra-rater reliability (κ = 0.79–0.93) [56] was more consistent than inter-rater reliability (ICC = 0.37 to 0.75) [49] (Table 5). Escolar et al. [55] evaluated both MMT and RQMS neurological impairment tests. Inter-rater reliability for MMT (ICC = 0.87) was reported for Escolar et al., [55] however consisted of a composite score of the strength of upper and lower limb muscle groups. Muscle strength scale definitions varied between studies (Table 6). Body positions and measurement methods were comparable to those used with HHD with the addition of smaller muscle groups, such as the ankle invertors and evertors (Table 6). Body positions and measurement methods were comparable to those used with HHD with the addition of smaller muscle groups, such as the ankle invertors and evertors (Table 6). The MMT in primary papers [49,55,56] did not require any equipment; therefore it is also a portable test. [14]

#### Charcot marie-tooth paediatric scale

The Charcot-Marie-Tooth Pediatric Scale (CMTPedS) was evaluated in one paper with children and young people with Charcot-Marie-Tooth (Table 3, Table 4). [45] The methodological quality of the reliability component of the paper on CMTPedS identified 75% of quality items using the Brink and Louw [6] criteria, (Table 3) yet was rated as poor using the COSMIN checklist [8] due to a sample size considered small (Table 4). The CMTPedS had very high reported inter-rater reliability (ICC = 0.95). [45] The CMTPedS score, however, was a composite score, including upper and lower limb test components with subsets of muscle strength and tactile sensitivity tests comprising 36% of items within the test. While this test has high portability, the need for other equipment, including HHD, [45] at an approximate cost of USD$2657 [45] in conjunction with the need for additional training reduces its overall clinical utility. [14]

#### ASIA impairment scale

The International Standards for Neurological Classification of Spinal Cord Injury American Spinal Injury Association (ASIA) scale was evaluated in one paper with children and young people with spinal cord injury (Table 3, Table 4). [57] The methodological quality of the of the paper on the ASIA scale identified 50% of quality items using the Brink and Louw [6] criteria, (Table 3) and was rated as poor using the COSMIN checklist [8] due to methodological flaws considered major (Table 4). The ASIA scale reported high intra-rater reliability (ICC = 0.71 to 0.98), with wide variation in 95% CI (0.23 to 0.99). [57] The ASIA impairment scale, is a composite score, including upper and lower limb test components with subsets of motor scores and tactile sensitivity tests (including pinprick and light touch). This test has high portability, without the need for other equipment, although requires some training. [57]

#### Richmond quantitative measurement system

Clinimetric evidence for the Richmond Quantitative Measurement System was identified in one paper in children and young people with cerebral palsy (Table 3, Table 4). [55] The methodological quality of the paper [55] identified 63% of items scored “yes” with the Brink and Louw critical appraisal tool, [6] but was rated as poor using the COSMIN checklist [8] (Table 3, Table 4). The Richmond Quantitative Measurement System had moderate to very high inter-rater reliability (ICC = 0.56 to 0.97) (Table 5). [55] This was determined in one study with poor methodological quality (Table 4). The Richmond quantitative measurement system requires equipment with specialised software and training requirements reducing its portability and clinical utility.

#### Standing heel rise

One study provided evidence on the reliability of the Standing Heel Rise [52] in children and young people with cerebral palsy (Table 3, Table 4). The methodological quality of this paper [52] showed 75% of the Brink and Louw [6] items were rated as “yes”, however the overall paper [52] was rated poor using the COSMIN checklist, [8] due to a sample size considered small (Table 4). Intra-rater reliability was very high (ICC = 0.84–0.99), [52] using the protocol by Van Vulpen et al. [52] This protocol was portable, however it involved the additional use of infra-red beams connected to a receiver which detected the heels lifting 1.7cm off the ground. There was no detail regarding training requirements or equipment costs for the SHR.

### Synthesis of evidence

A best evidence synthesis for each of the six neurological tests showed HHD had conflicting evidence on reliability for children with cerebral palsy, moderate inter-rater reliability for children with spina bifida and moderate intra-rater reliability for children with Duchenne’s muscular dystrophy (Table 7). MMT had conflicting evidence regarding intra-rater and inter-rater reliability in children with Duchenne’s muscular dystrophy and spina bifida respectively. Moderate evidence was found for the Charcot-Marie-Tooth Pediatric Scale [45] and the Standing Heel Rise. [52] These tests had consistent evidence across multiple studies and were published in papers higher in methodological quality, which resulted in greater evidence ratings despite small bodies of evidence (Table 7). Conflicting evidence on intra-rater reliability was found for both the motor and sensory constructs of the ASIA Impairment scale when used on children and young people with a SCI. The Richmond Quantitative Measurement Scale [55] had unknown evidence of inter-rater reliability due to the poor methodological quality of the published papers.

**Table 7:** Levels of evidence^a^ of lower limb strength measurements based on Brink and Louw^b^ methodological quality.

Neurological test or method	Diagnosis	Measurement property			
		Intra-rater reliability	Inter-rater reliability	Validity	Responsiveness
ASIA Impairment Scale	SCI	± Conflicting [57]	No evidence	No evidence	No evidence
CMTPedS	CMT	No evidence	++ Moderate [45]	No evidence	No evidence
HHD	CP	± Conflicting^c^ [46, 47, 51, 52, 54]	± Conflicting [53]	No evidence	No evidence
	SB	+ Limited [48]	++ Moderate [49]	No evidence	No evidence
	DMD	++ Moderate [50]	No evidence	No evidence	No evidence
MMT	SB	No evidence	± Conflicting [49]	No evidence	No evidence
	DMD	± Conflicting [55,56]	? Unknown [55]	No evidence	No evidence
RQMS	CP	No evidence	? Unknown [55]	No evidence	No evidence
SHR	CP	++ Moderate [52]	No evidence	No evidence	No evidence

CMTPedS, Charcot- Marie- Tooth Paediatric Scale; HHD, Hand held dynamometer; MMT, Manual muscle test; RQMS, Richmond Quantitative Measurement System; SHR, Standing heel rise; +++ or— = **strong evidence** with consistent findings from two or more good quality papers or one paper of excellent quality; ++ or— = **moderate evidence** with consistent findings from two or more fair quality papers or one paper of good quality; + or— = **limited evidence** with consistent findings from one paper of fair quality, ± = **conflicting evidence** with inconsistent findings from one or more papers of fair quality,? = **unknown evidence** with findings only from papers of poor quality, 0 = **no evidence**.

^a^Adapted from Terwee et al., [7] Dobson et al. [31] and Dekkers et al. [20]

^b^Methodological quality based on Brink and Louw et al.’s [6] critical appraisal tool using an arbitrary grades based on the percentage of “yes” responses for applicable items. Arbitrary grades: <40% = Poor, 40%-59% = Fair, 60% - 79% = Good, >80% = Excellent.

^c^Both Taylor et al. [51] and Van Vulpen et al. [52] report “test-retest reliability”, however their measurement characteristics (S4 Table) fit the definition of intra-rater reliability defined as defined in Table 1.

## Discussion

This is the first study to systematically identify clinimetric evidence on lower limb neurological impairment tests used on children and young people across a range of neurological disorders. Evidence of reliability was the only identified clinimetric property for six of the identified 21 neurological tests, demonstrating the paucity of evidence for neurological impairment testing. Clinimetric evidence for tactile sensitivity was identified in two primary papers [45,57] containing composite measures. However, tactile sensitivity evidence could only be extrapolated from one primary paper. [57] The limited to moderate body of evidence on reliability of lower limb muscle strength tests and composite tests including subsets of tactile sensitivity and muscle strength, highlights the lack of robust clinimetric evidence for neurological tests in a paediatric population with a lower limb neurological condition. This is further illustrated by no available clinimetric evidence for deep tendon reflex tests despite tests existing to evaluate this construct. Despite the limited and conflicting evidence, hand held dynamometry, manual muscle testing and the standing heel rise, may provide a starting point from which to develop high quality clinimetric studies that evaluate specific testing protocols for children and young people with a neurological condition.

Existing clinimetric evidence must be interpreted in conjunction with the methodological quality of the paper. [6,44] Ten of the 13 included papers in this study had greater than 60% of “yes” items for methodological quality using the Brink and Louw method, [6] compared with the COSMIN checklist grading 12 of 13 papers in this study with a ‘*poor quality*’ due to small sample sizes (less than 30). [8,44] The small sample size of children within primary papers has previously been highlighted as a potential limitation. [1,20] Benfer et al. [41] argued that smaller sample sizes are common in paediatrics, yet these studies may have adequate power to support their small sample. [49] Similar systematic reviews have used a ‘second-worst’ method with a modified COSMIN to combat this issue, however this technique has not been validated. [20,22,23,41,58]

The Brink and Louw [6] critical appraisal tool highlighted specific methodological flaws of included papers, the most prevalent being the lack of reported stability for a child’s condition across testing sessions. Ensuring the stability of a child’s condition means any identified differences are due to measurement error [16] and not changes in their condition. [59–62] The time between testing sessions should be considered relative to the underlying diagnosis to ensure there is no expected change or fatigue. The stability results for a participants neurological condition reported in five [45,49,50,52,54] of the six primary papers, [45,49,50,52,54,57] (Item 8, Table 3) should be interpreted with caution, as these papers did not state whether the time frame between sessions was appropriate for their population-group or if the child or carer believed there to be no change in the child’s status between testing sessions. Reliability cannot be inferred without measuring whether a child’s condition is stable across testing sessions. [16] Reliability coefficients in primary papers could therefore be lower than reported due to the absence of stability measures.

Clinimetric evidence was only identified for muscle strength tests, and was limited to evidence on reliability. Reliability has also been the primary identified clinimetric property in a similar review of upper limb tests of muscle strength in children with cerebral palsy. [20] The paucity of additional primary papers since Mulder-Brouwer et al’s [23] study also highlights the lack of an increase in the body of literature since 2013. The inconsistent reporting of evidence on reliability in the identified neurological tests makes interpretation and research translation difficult. Reliability was quantified using ICC or weighted kappa for 14 of the 15 primary papers, however the use of different measurement protocols made it difficult to draw conclusions and prevented a meta-analysis. Mahony et al. [49] calculated an ICC from ordinal data instead of the appropriate weighted kappa confounding the interpretation of their reliability values.

Reliability is defined as a measure that is consistent and free from random or systematic error (Table 1). [16,63] Additional statistics, such as the 95% CI and systematic error of measurement (SEM), aid in the interpretation of the test’s reliability. [59,63] Wide CIs in the few primary papers reporting 95% CI, indicated variation in this measurement property in children. [47,57] The SEM provides clinicians and researchers with information on the systematic and random error of a patient’s score that is not attributed to true change. [54,63] Reliability for neurological tests reported in the seven papers [45,48,50,51,55–57] that did not report SEM should therefore be interpreted with caution. Comparisons of SEM, where reported, could not be made between primary papers in this study due to different units of measurement, muscle groups tested and protocols used. A standardised measurement protocol would therefore provide the same units for SEM to aid in reporting random error [16] and assist in synthesising results from multiple primary studies.

Results of this study indicate that the same clinician should perform each neurological test due to consistently higher intra-rater reliability coefficients compared to inter-rater reliability coefficients (Fig 2). All clinicians who used the neurological tests in the included papers were reported to have six or more years of clinical experience. Without reporting the clinician’s experience in using the neurological test, or comparing to clinicians with less than six years of clinical experience, the effect clinician experience has on the outcome of neurological testing on children and young people with a neurological condition is unknown. [16] A recent reliability study of manual muscle testing in children and young people with spina bifida suggested experienced clinicians should assist in training novice clinicians to improve measurement reliability. Tan et al.’s 2016 (in press) [64] study reported an overall weighted kappa of 0.95 (CI 0.94–0.96) for MMT using the Daniel’s and Worthingham’s protocol, yet the methodological quality would have been graded as poor using the COSMIN checklist due to a small sample size. [8,64]

Manual muscle testing is typically recommended in weaker muscles, with equal or less than gravity strength, [54,64] yet this test becomes more variable when the clinician needs to apply increasing amounts of resistance. (i.e. grade IV to V) [65] Clinicians should use the make method when performing hand held dynamometry and manual muscle testing as the larger body of evidence and increased reliability (Fig 2) supports this method compared to the break test. Evaluation of the ankle plantarflexors was an exception to this finding. [53] The ankle plantarflexors are known to be a strong muscle group that acts upon a short lever arm, making it challenging for clinicians to apply sufficient manual resistance for muscle testing. [66–69] This often limits muscle testing of ankle plantarflexors group to the relative strength of the clinician performing the test. [70] However, clinician strength was not a reported variable in the primary papers. The moderate evidence for standing heel rise test may suggest using this as an alternative test for measuring plantarflexion muscle strength in ambulatory children.

Inconsistent muscle strength testing methods between the primary papers confirms that a standardised test protocol for muscle strength testing does not exist. There is also wide variability in grading scales when using MMT, with four different scales reported in the three MMT papers [49,55,56] with clinimetric evidence and the motor subscale of the ASIA scale [57]. The conflicting evidence on reliability for hand held dynamometry found in papers of fair quality means additional high quality research, using a standardised measurement protocol, is required to make recommendations. Consensus between clinicians on a standard protocol is recommended prior to further clinimetric testing. Without clinimetric evidence, lower limb rehabilitation trials for children and young people are at risk of bias due to the use of neurological tests with unknown clinimetric properties. [71]

Reliability is only one of many clinimetric properties, which include validity, responsiveness and clinical utility, [7,14] The Charcot-Marie-Tooth Pediatric Scale (CMTPedS) [45] has clinimetric evidence of both reliability and validity, however the age range of participants differed between those who participated in the reliability and validity studies. The evidence on reliability, which was included in this study, was for children and young people aged 5–15 years. However, evidence on the validity of the CMTPedS was for children and young people aged 3–20 years. [45] Evidence of validity for the CMTPedS could not be included in this study due to the age ranges of participants exceeding 18 years of age, as per the exclusion criteria. Without clinimetric evidence presented for different age groups, it is unclear whether the validity evidence for the CMTPedS [45] is specific to the paediatric population. Clinimetric evidence for the ASIA scale [57] was included in this study by extrapolating data for children and young people aged 4–15, while the 16–21 year old age group data were not included in this study.

Currently there is no universally accepted definition of the upper age limits [25] for a paediatric population from other paediatric systematic reviews. [20,22,30,39,58] A definition of paediatrics as children less than 18 years was used in this study to align with previous systematic reviews with a paediatric population [1,20–22,39] and Medical Subject Headings definitions for a targeted search strategy. [27] The comprehensive search strategy used in this study [24,26,27] ensured the identification of lower limb impairment neurological tests that were specific to children and young people with a neurological condition. [42] Future studies may broaden the paediatric age range up to 22 years of age as suggested by Clark et al. [25] Until future research supports this upper age limit, papers should report evidence for different paediatric age ranges to allow for greater research translation. [25,72]

In contrast to previous reviews [9,20,23,30,31,41], this study covers a broad paediatric age range and multiple neurological conditions. Recommendations for a clinimetrically-sound neurological test require a standardised test protocol with population-specific evidence, as clinimetric properties from other populations are not inherently transferable. [13,14,42] The majority of papers identified in this review had clinimetric evidence of neurological impairment tests used on children with cerebral palsy, which likely reflects cerebral palsy as the most prevalent paediatric neurological condition with motor and sensory impairment. [73]

This study was limited to three components of a neurological examination at the ‘body function and structures’ level of the ICF-CY as other neurological impairment tests such as spasticity are dependent on the diagnosis of the child. [4] For a comprehensive neurological examination other components of a neurological examination should be included, such as measures from the ‘activity’ and ‘participation’ levels of the ICF-CY. [4,74] Selection of these neurological tests will be dependent on the diagnosis of the child. Limited evidence for clinimetrically-sound measures of ‘activity’ for children and young people with a neurological condition have been found, [22,58] demonstrating a similar shortage of high-quality studies in these constructs.

Synthesising best evidence, through combining a consistent body of clinimetric evidence with robust methodological qualities, can guide clinicians and researchers to select appropriate paediatric-specific lower limb neurological tests. [7,31] Guidance on best evidence of clinimetrically-sound measures cannot be made with reliability evidence alone. Without evidence of reliability, validity, responsiveness and clinical utility, recommendations to clinicians for neurological tests can only be made with caution until further clinimetric evaluation can be used to support best practice. [7,75]

## Conclusion

There is a lack of robust clinimetric evidence on neurological impairment tests to use on children and young people with a lower limb neurological condition. Clinimetric evidence was only found on the reliability of neurological impairment tests evaluating muscle strength. Performing standardised testing protocols, such as the make method, with manual or belt stabilisation in a stable population-specific group, are recommended as a starting point for further clinimetric studies. In the absence of clinimetrically-sound neurological tests, clinicians should use the best available evidence. Without clinimetrically-sound neurological tests it is difficult for clinicians and researchers to select and perform a test in clinical practice, which becomes increasingly complex when requiring a combination of these tests for a thorough neurological examination. High quality, population-specific studies are required to provide a strong body of clinimetric evidence for clinicians and researchers to make future recommendations for use of a neurological examination in clinical practice and research.

## Supporting information

S1 Table CINAHL search terms used to identify lower limb neurological tests in children. (DOCX)

S2 Table CINAHL search terms used to identify clinimetric properties for Achilles reflex in children. (DOCX)

S3 Table Neurological test names for children and adolescents identified by the search strategies, and papers on their clinimetric properties. (DOCX)

S4 Table Characteristics of included clinimetric papers for neurological tests used on children and young people with a neurological condition. (DOCX)

S5 Table PRISMA checklist. (DOC)
