# Supplementary material for: Clinimetric properties of lower limb neurological impairment tests for children and young people with a neurological condition: A systematic review
Source: PLoS One. 2017 Jul 3;12(7):e0180031. doi: 10.1371/journal.pone.0180031 (PMC5495217; doi:10.1371/journal.pone.0180031)
Supplement: S1 Table — (DOCX) [file pone.0180031.s001.docx]

S1 Table: CINAHL search terms used to identify lower limb neurological tests in children

|  | (MH “Lower Extremity+”) |
| --- | --- |
|  | TI lower extremity OR AB lower extremity OR TI lower limb OR AB lower extremity OR TI leg OR AB leg |
|  | TI neuro* test* OR AB neuro* test* OR TI neuro* assess* OR AB neuro* assess* OR TI neuro* exam* OR AB neuro* exam* OR TI physical assess* OR AB physical assess* OR TI physical exam* OR AB physical exam* OR TI physical test* OR AB physical test* |
|  | (MH "Neurologic Examination") OR (MH "Physical Examination") |
|  | TI sensation OR AB sensation OR TI reflex* OR AB reflex* OR TI muscle strength OR AB muscle strength |
|  | (MH "Sensation") OR (MH "Reflex") OR (MH "Muscle Strength") |
|  | TI child* OR AB child* OR TI paediatric* OR AB paediatric* OR TI pediatric* OR AB pediatric* |
|  | (MH "Child") OR (MH "Adolescence") OR (MH "Child, Preschool") |
|  | (1 OR 2) AND (3 OR 4) AND (5 OR 6) AND (7 OR 8) |
|  | Published Date: 19850101-; English Language; Peer Reviewed; Age Groups: Child, Preschool: 2-5 years, Child: 6-12 years, Adolescent: 13-18 years |

CINAHL = Cumulative Index to Nursing and Allied Health Literature Database

MH = Medical subject heading (MeSH), + indicates ‘exploded’ MeSH terms, TI = title, AB = Abstract
