# Supplementary material for: Clinimetric properties of lower limb neurological impairment tests for children and young people with a neurological condition: A systematic review
Source: PLoS One. 2017 Jul 3;12(7):e0180031. doi: 10.1371/journal.pone.0180031 (PMC5495217; doi:10.1371/journal.pone.0180031)
Supplement: S2 Table — (DOCX) [file pone.0180031.s002.docx]

**S2 Table:** CINAHL search terms used to identify clinimetric properties for achilles reflex in children

| Neurological measure search | 1. (AB "lower extremity" OR TI "lower extremity" OR MH “lower extremity+” OR AB leg OR TI leg OR MH leg+) |
| --- | --- |
|  | 1. AB Power OR AB "Muscle strength" OR AB Resistance OR AB Strength OR AB sensation OR AB sensitivity OR AB tactile OR AB somatosens* OR AB reflex* OR TI Power OR TI "Muscle strength" OR TI Resistance OR TI Strength OR TI sensation OR TI sensitivity OR TI tactile OR TI somatosens* OR TI reflex* |
| Population search | 1. (MH Child OR MH adolescent OR MH “child, preschool” OR AB pediatric OR AB paediatric) |
| Neurological test search | 1. (AB "achilles reflex" OR TI "achilles reflex" OR AB "ankle jerk" OR TI "ankle jerk" OR AB "achillean reflex" OR TI "achillean reflex") |
| Measurement property filter | 1. (MW instrumentation OR MW methods OR PT “Validation Studies” OR PT “Comparative Study” OR MH “psychometrics” OR TI psychometr* OR AB psychometr* OR TX clinimetr* OR TX clinometr* OR MH “outcome assessment (health care)” OR TI “outcome assessment” OR AB “outcome assessment” OR TX “outcome measure*” OR MH “observer variation” OR TI “observer variation” OR AB “observer variation” OR MH “Health Status Indicators” OR MH “reproducibility of results” OR TI reproducib* OR AB reproducib* OR MH “discriminant analysis” OR TI reliab* OR AB reliab* OR TI unreliab* OR AB unreliab* OR TI valid* OR AB valid* OR TI coefficient OR AB coefficient OR TI homogeneity OR AB homogeneity OR TI homogeneous OR AB homogeneous OR TI “internal consistency” OR AB “internal consistency” OR ((TI cronbach*OR AB cronbach*) AND (TI alpha OR AB alpha OR TI alphas AB alphas)) OR ((TI item OR AB item) AND (TI correlation* OR AB correlation* OR TI selection* OR AB selection* OR TI reduction* OR AB reduction*)) OR TI agreement OR AB agreement OR TI precision OR AB precision OR TI imprecision OR AB imprecision OR TI “precise values” OR AB “precise values” OR TI test–retest OR AB test–retest OR ((TI test OR AB test) AND (TI retest OR AB retest)) OR ((TI reliab* OR AB reliab*) AND (TI test OR AB test OR TI retest OR AB retest)) OR TI stability OR AB stability OR TI interrater OR AB interrater OR TI inter-rater OR AB inter-rater OR TI intrarater OR AB intrarater OR TI intra-rater OR AB intra-rater OR TI intertester OR AB intertester OR TI inter-tester OR inter-tester OR TI intratester OR AB intratester OR TI intra-tester OR AB intra-tester OR TI interobserver OR AB interobserver OR TI inter-observer OR AB inter-observer OR TI intraobserver OR AB intraobserver OR TI intraobserver OR AB intraobserver OR TI intertechnician OR AB intertechnician OR TI inter-technician OR AB inter-technician OR TI intratechnician OR AB intratechnician OR TI intra-technician OR AB intra-technician OR TI interexaminer OR AB interexaminer OR TI inter-examiner OR AB inter-examiner OR TI intraexaminer OR AB intraexaminer OR TI intra-examiner OR AB intra-examiner OR TI interassay OR AB interassay OR TI inter-assay OR AB inter-assay OR TI intraassay OR AB intraassay OR TI intra-assay OR AB intra-assay OR TI interindividual OR AB interindividual OR TI inter-individual OR AB inter-individual OR TI intraindividual OR AB intraindividual OR TI intra-individual OR AB intra-individual OR TI interparticipant OR AB interparticipant OR TI inter-participant OR AB inter-participant OR TI intraparticipant OR AB intraparticipant OR TI intra-participant OR AB intra-participant OR TI kappa OR AB kappa OR TI kappa’s OR AB kappa’s OR TI kappas OR AB kappas OR TI repeatab* OR AB repeatab* OR ((TI replicab*OR AB replicab* OR TI repeated OR AB repeated) AND (TI measure OR AB measure OR TI measures OR AB measures OR TI findings OR AB findings OR TI result OR AB result OR TI results OR AB results OR TI test AB test OR TI tests OR AB tests)) OR TI generaliza* OR AB generaliza* OR TI generalisa* OR AB generalisa* OR TI concordance OR AB concordance OR ((TI intraclass OR AB intraclass) AND (TI correlation* OR AB correlation*)) OR TI discriminative OR AB discriminative OR TI “known group” OR AB “known group” OR TI factor analysis OR AB factor analysis OR TI “factor analyses” OR AB “factor analyses” OR TI dimension* OR AB dimension* OR TI subscale* OR AB subscale* OR ((TI multitrait OR AB multitrait) AND (TI scaling OR AB scaling) AND (TI analysis OR AB analysis OR TI analyses OR AB analyses)) OR TI “item discriminant” OR AB “item discriminant” OR TI “interscale correlation*” OR AB “interscale correlation*” OR TI error OR AB error OR TI errors OR AB errors OR TI “individual variability” OR AB “individual variability” OR ((TI variability OR AB variability) AND (TI analysis OR AB analysis OR TI values OR AB values)) OR ((TI uncertainty OR AB uncertainty) AND (TI measurement OR AB measurement OR TI measuring OR AB measuring)) OR TI “standard error of measurement” OR AB “standard error of measurement” OR TI sensitiv* OR AB sensitiv* OR TI responsive* OR AB responsive* OR ((TI minimal OR AB minimal OR TI minimally OR AB minimally OR TI clinical OR AB clinical OR TI clinically OR AB clinically) AND (TI important OR AB important OR TI significant OR AB significant OR TI detectable OR AB detectable) AND (TI change OR AB change OR TI difference OR AB difference)) OR ((TI small* OR AB small*) AND (TI real OR AB real OR TI detectable OR AB detectable) AND (TI change OR AB change OR TI difference OR AB difference)) OR TI “meaningful change” OR AB “meaningful change” OR TI “ceiling effect” OR AB “ceiling effect” OR TI “floor effect” OR AB “floor effect” OR TI “Item response model” OR AB “Item response model” OR TI IRT OR AB IRT OR TI Rasch OR AB Rasch OR TI “Differential item functioning” OR AB “Differential item functioning” OR TI DIF OR AB DIF OR TI “computer adaptive testing” OR AB “computer adaptive testing” OR TI “item bank” OR AB “item bank” OR TI “cross-cultural equivalence” OR AB “cross-cultural equivalence”) |
|  | 1. 1 AND 2 AND 3 AND 4 AND 5 |

Measurement property search adapted from Terwee et al 2009

CINAHL = Cumulative Index to Nursing and Allied Health Literature Database

AB = Abstract, TI = Title, MH = Medical subject heading, MW = Word in subject heading, PT = publication type
