# Supplementary material for: Clinimetric properties of lower limb neurological impairment tests for children and young people with a neurological condition: A systematic review
Source: PLoS One. 2017 Jul 3;12(7):e0180031. doi: 10.1371/journal.pone.0180031 (PMC5495217; doi:10.1371/journal.pone.0180031)
Supplement: S3 Table — (DOCX) [file pone.0180031.s003.docx]

**S3 Table. Neurological test names for children and adolescents identified by the search strategies, and papers on their clinimetric properties.**

|  | **Neurological test** | **Construct** | **Description** | **Papers evaluating the lower limb of children and young people with a neurological condition (Phase ^[1^)** | **Reports on clinimetric properties**  **(Phase ^[2^)** |
| --- | --- | --- | --- | --- | --- |
|  | Deep tendon reflex (Including: Achilles reflex, Ankle jerk, patella reflex, knee jerk) | Reflex | A tendon hammer is usually used to apply a rapid force to a tendon while it is under stretch. The reflex movement is usually quantified on a three-point scale. | Auregan et al. ^[76];^ Demir et al.^[77];^ Dogan et al.^[78];^ Doymaz et al.^[79];^ Dunin-Wasowicz et al.^[80];^ Eljebbouri et al.^[81^], Huang et al.^[82];^ Karadaş et al.^[83];^ Kobayashi et al.^[84];^ Maytal et al.^[85];^ Mancias et al.^[86];^ McLaughlin et al.^[87];^ Mridha et al.^[88];^ Mutoh et al.^[89];^ Nakano et al.^[90];^ Nevo et al.^[91];^ O’Sullivan et al. ^[92];^ Padua et al. ^[93^ Roizen et al.^[94];^ Roy et al.^[95];^ Salih et al. ^[96];^ Saroyan et al. ^[97];^ Saxena et al.^[98];^ Tullu et al.^[99];^ Udayashankar et al.^[100];^ Wan et al.^[101];^ Wang et al.^[102];^ Yagev et al.^[103];^ Ziv et al. ^[104]^ |  |
|  | Electromyometer | Muscle strength | A small displacement transducer is mounted in a measuring head. The signal is amplified and recorded in a battery operated recording unit. A force applied to the measurement head is usually measured in kg. The electromyometer may be a hand held device. | Kroksmark et al.^[105];^ Scott et al.^[106^, van der Linden et al.^[107]^ |  |
|  | Fixed / isometric dynamometer | Muscle strength | An isometric force (i.e. a force against an immovable object where the joint angle is constant) is measured using a dynamometer that is mounted on a static device. Usually measured in N/kg or kg. | Brouwer et al.^[108];^ Fowler et al.^[109];^ Refshauge et al.^[110];^ Seniorou et al.^[111]^ |  |
|  | Hand held dynamometer (HHD)  (Including: Nicholas manual muscle tester) | Muscle strength | A portable dynamometer that is held in the hand. The dynamometer typically consists of multiple strain gauges that act on a transducer head as a force is applied. The method of force application is either the **“make” test**, where the child slowly matches the applied force, or the **“break” test**, where the clinician attempts to overcome the child’s maximal force. Usually measured in N/kg or kg. | Hoesl et al.^[112];^ Liao et al.^[113];^ Lidbeck ^[114];^ Mahony et al.^[48];^ Manzur et al.^[115];^ Nevo et al.^[91];^ Pagliano et al.^[116];^ Reyes et al.^[117];^ Rose et al.^[118];^ Schoenmakers et al.^[119];^ Scholtes et al.^[120];^ Scholtes et al.^[121];^ Seniorou et al.^[111];^ Stuberg et al.^[50];^ Tedla et al.^[122];^ Verschuren et al.^[123]^ | Berry et al. ^[46]^ ; Crompton et al.^[47]^ ; Effgen et al.^[48]^ ; Mahony et al. ^[49]^; Stuberg et al. ^[50]^; Taylor et al. ^[51]^; Van Vulpen et al. ^[52]^; Verschuren et al. ^[53]^; Willemse et al.^[54]^ |
|  | Isokinetic dynamometer | Muscle strength | A device that consists of a transducer mounted to a lever arm. The axis of the lever arm is adjusted to the level of the child’s joint axis. The distal lever arm is strapped to the distal lower limb and children are stabilised at the proximal thigh and trunk. The distance from the force transducer to the axis of rotation is recorded. The dynamometer measures a concentric contraction, usually knee flexion and extension. The force is applied throughout the joint range of motion at a set angular velocity. Usually measured in Nm. | Arpin et al.^[124];^ Chen et al.^[125];^ Engsberg et al.^[126];^ Fowler et al.^[109];^ Vershuren et al.^[52]^ | Ayalon et al. ^[126]^; Berg-Emons et al. ^[127]^ |
|  | Manual muscle test (including British Medical Research Council scale / Daniel's and Worthinghams scale, Oxford scale, Mendall and Florence protocol, Lovett scale) | Muscle strength | Child is asked to move about a single joint. The test progresses from gravity eliminated positions, to against gravity, before manual application of moderate resistance to the distal end of a joint, and then manually applying a maximal resistance. The method of force application is either the **“make” test**, where the child slowly matches the applied force, or the **“break” test**, where the clinician tries to overcome the individual’s maximal force. Muscle grades are given using an ordinal scale, which is either a six-point scale from 0-5 or a twelve-point scale if half points are given. | Brown et al.^[129];^ Demir et al.^[77];^ Dogan et al.^[78];^ Geerdink et al.^[130];^ Gualdi et al.^[131];^ Haberlova et al.^[132];^ Huddleston et al.^[133];^ Tshala-Katumbay et al.^[134];^ Kaya et al. ^[135];^ Komarowska et al.^[136];^ Labruyere et al.^[137];^ Langerak et al.^[138];^ Lin et al.^[139];^ Mahony et al.^[49];^ Manzur et al.^[115];^ Padua et al.^[93];^ Roy et al.^[95];^ Schoenmakers et al.^[119];^ Scott et al.^[106];^ Wehby et al.^[141]^ | Mahony et al. ^[49]^; Escolar et al. ^[55]^; Florence et al. ^[56]^ |
|  | Maximum isometric contraction  / Maximum voluntary contraction | Muscle strength | The maximal force a muscle can produce against an immovable object. This may involve quantitative muscle testing or manual muscle testing methods. | Brouwer et al.^[108];^ Reyes et al.^[117];^ Scott et al.^[106];^ Seniorou et al.^[111]^ | Escolar et al. ^[55]^ |
|  | Quantitative Muscle Testing | Muscle strength | Includes any measurement device for continuous measuring of muscle strength (including hand held dynamometer, Richmond Quantitative Measurement System, myometer, strain gauge, spring scale goniometers, electromyometer). Usually muscle force is measured in N or N/kg or kg. Does not include ordinal measurement scales such as those used in manual muscle testing. |  |  |
|  | Repetition maximum | Muscle strength | The force required to overcome a maximum weight, a pre-set number of times, often a maximum of one, six, or eight repetitions. | Scholtes et al.^[120];^ Scholtes et al.^[121]^ |  |
|  | Spring scale / balance (including spring scale goniometer) | Muscle strength | One end of the spring scale is usually mounted to a static device. An opposing force is applied to the opposite end of the spring scale to stretch the internal spring. The distance the spring is extended is usually converted to an equivalent reading of N or kg. | Refshauge et al.^[110];^ Verschuren et al.^[123]^ |  |
|  | American Spinal Cord Injury Association (ASIA) Impairment Scale (including International Standard for Neurological Classification of Spinal Cord Injury) | Muscle strength / Sensation | A composite score of strength (0-5 point scale) and sensation (0-2 point scale) for major muscle groups and neurological levels of the upper and lower limb. | Furlan et al.^[142];^ Pierce et al.^[143]^ | Mulcahey et al, ^[57]^ |
|  | Charcot-Marie-Tooth Neuropathy Score (CMTNS) | Muscle strength / Sensation | A composite score comprising of symptoms, signs, and neurophysiological tests of the upper and lower limbs quantified on a five-point scale. Parameters include sensory symptoms, motor symptoms, pinprick sensibility, vibration, strength, ulnar compound muscle action potential, and radial sensory action potential. | Haberlova et al.^[132];^ Pagliano et al.^[116]^ |  |
|  | Charcot-Marie-Tooth Pediatric Scale (CMTPedS) | Muscle strength / Sensation | A composite score comprising of hand dexterity, strength, sensation, balance and motor function of the arms and legs quantified using a five-point scale. Parameters include: functional dexterity test, nine-hole peg test, hand grip, foot plantar flexion, foot dorsiflexion, pinprick, vibration, Bruininks Osertsky test, gait, long jump, and six minute walk test. | Acsadi et al.^[144];^ Burns et al.^[145]^ | Burns et al. ^[45]^ |
|  | Severity scale for clinical assessment of tethered spinal cord syndrome | Muscle strength / Tactile Sensitivity / Deep Tendon Reflexes | A composite score for each limb involving measures of gait (0-4 scale), bowel/bladder history (0-4 scale), sensory-pinprick (0-2 scale), lower limb strength (manual muscle testing with 0-5 scale), and deep tendon reflexes (0-4+ scale). Lower scores indicate increased severity, whereas the maximum scores indicate no clinical signs or symptoms. | Roy et al.^[95]^ |  |
|  | Fine touch /  Light touch | Tactile Sensitivity | Light application of a stimulus to the skin. The stimulus is often a cotton tip, and application is either initial skin contact or sweeping across the skin. Sensation is usually quantified either through comparison to a point elsewhere on the body or using an eleven-point scale (0-10). | Furan et al.^[133];^ Huddleston et al.^[124];^ Maytal et al.^[78];^ Udayashankar et al.^[93];^ Wehby et al.^[132];^ Yagev et al.^[96]^ |  |
|  | Pinprick | Tactile Sensitivity | Application of a sharp stimulus to the skin. The stimulus may involve the sharp end of a paper clip, or a manufactured device such as the neurotip ©. Application involves depression of the skin, but not to the point of skin damage. Sensation may be quantified by comparing to a point elsewhere on the body or using an eleven-point scale (0-10) or a 3-point scale (0-2). | Mancias et al.^[79];^ Maytal et al.^[78];^ Roy et al.^[95];^ Ziv et al.^[97];^ Yagev et al.^[96]^ |  |
|  | Pinwheel | Tactile Sensitivity | Application of a series of evenly spaced sharp stimuli through the use of a pinwheel and quantifying either through comparison to the other side or on an eleven-point scale. | Wehby et al.^[132]^ |  |
|  | Position sense  / Proprioception | Tactile Sensitivity | A therapist moves a lower limb join into a position without the child seeing. The child describes the direction of movement and or position of the limb based on what they felt. | Karadas et al.^[8]^; Mutoh et al.^[82];^ Salih et al.^[89];^ Wehby et al.^[133]^ |  |
|  | Temperature | Tactile Sensitivity | Differentiation between hot (less than 35^o^C) and cold (above 4oC) temperatures. Usually quantified as a correct or incorrect response. | Imtaka et al.^[135];^ Komarowska et al.^[127];^ Mutoh et al.^[82];^ Udayashankar et al.^[93]^ |  |
|  | Vibration | Tactile Sensitivity | Application of a vibrating tuning fork (usually a 158Hz or C tuning fork) on a bony prominence. Test usually begins distally and progresses proximally and is quantified on whether sensation is or is not perceived. A timed vibratory test may be used to quantify the length of time a child perceives vibration. | Karadaş et al.^[76];^ Moody et al.^[138];^ Nakano et al.^[83];^ Salih et al.^[89];^ Udayashankar et al.^[93]^ |  |
|  | Standing heel rise test | Muscle strength | A child stands on one or both legs and is requested to maximally plantarflex their ankles so that they shift their weight up onto their toes. Examiners may allow children to lightly touch examiners hands with one finger for balance. The test is quantified by the number of repetitions achieved before the child is unable to achieve maximal plantarflexion, leans on examiner, bends their knees, or asks to stop. |  | Van Vulpen et al. ^[52]^ |

45. Burns J, Ouvrier R, Estilow T, et al. Validation of the Charcot-Marie-Tooth disease paediatric scale as an outcome measure of disability. Ann Neurol 2012; 71: 642-652.

46. Berry ET. Intrasession and intersession reliability of handheld dynamometry in children with cerebral palsy. Pediatr Phys Ther 2004; 16: 191-198.

47. Crompton J, Galea MP, Phillips B. Hand-held dynamometry for muscle strength measurement in children with cerebral palsy. Dev Med Child Neurol 2007; 49: 106-111.

48. Effgen SK, Brown DA. Long-term stability of hand-held dynamometric measurements in children who have myelomeningocele. Phys Ther 1992; 72: 458-465.

49. Mahony K, Hunt A, Daley D, et al. Inter-tester reliability and precision of manual muscle testing and hand-held dynamometry in lower limb muscles of children with spina bifida. Physical & Occupational Therapy in Paediatrics 2009; 29: 44-59.

50. Stuberg WA, Metcalf WK. Reliability of quantitative muscle testing in healthy children and in children with Duchenne muscular dystrophy using a hand-held dynamometer. Phys Ther 1988; 68: 977-982.

51. Taylor NF, Dodd KJ, Graham HK. Test-retest reliability of hand-held dynamometric strength testing in young people with cerebral palsy. Arch Phys Med Rehabil 2004; 85: 77-80.

52. Van Vulpen LF, De Groot S, Becher JG, et al. Feasibility and test-retest reliability of measuring lower‑limb strength in young children with cerebral palsy. Eur J Phys Rehabil Med 2013; 49: 803-813.

53. Verschuren O, Ketelaar M, Takken T, et al. Reliability of hand-held dynamometry and functional strength tests for the lower extremity in children with cerebral palsy. Disabil Rehabil 2008; 30: 1358-1366.

54. Willemse L, Brehm MA, Scholtes VA, et al. Reliability of Isometric Lower-Extremity Muscle Strength Measurements in Children With Cerebral Palsy: Implications for Measurement Design. Phys Ther 2013; 93: 935-941.

55. Escolar D, Henricson E, Mayhew J, et al. Clinical evaluator reliability for quantitative and manual muscle testing measures of strength in children. Muscle Nerve 2001; 24: 787-793.

56. Florence JM, Pandya S, King WM, et al. Intrarater reliability of manual muscle test (Medical Research Council scale) grades in Duchenne's muscular dystrophy. Phys Ther 1992; 72: 115-122.

57. Mulcahey M, Gaughan J, Betz R, Johansen K. The International Standards for Neurological Classification of Spinal Cord Injury: reliability of data when applied to children and youths. Spinal Cord. 2007;45:452-459.

76. Aurégan JC, Odent T, Zerah M, et al. Surgical treatment of a 180° thoracolumbar fixed kyphosis in a young achondroplastic patient: a one-stage "in situ" combined fusion and spinal cord translocation. Eur Spine J 2010; 19: 1807-1811

77. Demir Y, Aras B, Guzelkucnulluk U, et al. Lumbar disc herniation in an 11-year-old gymnastic player. Ann Phys Rehabil Med 2014; 57: e274.

78. Dogan I, Guner E, Kahilogullari G, Unlu A. A rare and unexpected clinical progress on a primary extradural spinal cyst hidatic-A case report. Childs Nerv Syst 2014; 30: 791.

79. Doymaz S, Sagy M, Schneider J. Nontraumatic epidural hematoma resulting in paraplegia in a teenage boy with cholestasis. Crit Care Med 2010; 38: A267.

80. Dunin-Wasowicz D, Jurkiewicz E, Jozwiak S. Coexistence of Guillain-Barre syndrome and ADEM in patient with ovarium teratoma-case report. Eur J Paediatr Neurol 2009; 13: S45-S46.

81. Eljebbouri B, Gazzaz M, Akhaddar A, et al. Paediatric intramedullary schwannoma without neurofibromatosis: Case report. Acta Med Iran 2013; 51: 727-729.

82. Huang PY, Lee SM. A case of acute myopathy due to a suspected Taiwan banded krait bite. Dev Med Child Neurol 2012; 54: 175.

83. Karadaş O, Öztürk B, Pekdal HI, Odabaiş Z. Chronic progressive monomelic sensory neuropathy of the right lower extremity in a child. Clin Neurol Neurosurg 2012; 114: 412-413.

84. Kobayashi S, Takahashi J, Sakashita K, et al. Ewing sarcoma of the thoracic epidural space in a young child. Eur Spine J 2013; 22: 373-379.

85. Maytal J, Wind E, Bierman F, et al. Acute myelopathy. Children's Hospital Quarterly 1995; 7: 241-244.

86. Mancias P, Krill J, Ketcham M, Bhattacharjee M. A child with progressive lower extremity weakness and a "twist". J Clin Neuromuscul Dis 2013; 14: 149-150.

87. McLaughlin JF, Bjornson KF, Astley SJ, et al. The role of selective dorsal rhizotomy in cerebral palsy: critical evaluation of a prospective clinical series. Dev Med Child Neurol 1994; 36: 755-769

88. Mridha AR, Sharma MC, Sarkar C, et al. Myxopapillary ependymoma of lumbosacral region with metastasis to both cerebellopontine angles: Report of a rare case. Childs Nerv Syst 2007; 23: 1209-1213

89. Mutoh K, Okuno T, Ito M, et al. Somatosensory evoked potentials after posterior tibial nerve stimulation in focal spinal cord diseases. Pediatr Neurol 1991; 7: 326-332.

90. Nakano S, Ohnishi A, Yamamoto T, et al. A case of hereditary motor and sensory neuropathy of neuronal type with retardation of motor development. Rinsho Shinkeigaku. 1990; 30: 448-451.

91. Nevo Y, Pestronk A, Lopate G, Carroll SL. Neuropathy of metachromatic leukodystrophy: Improvement with immunomodulation. Pediatr Neurol 1996; 15: 237-239.

92. O’Sullivan R, Kiernan D, Walsh M, et al. Characterisation of the patellar tendon reflex in cerebral palsy children using motion analysis. Ir J Med Sci 2015; 1971-:1-5.

93. Padua L, Rendeli C, Ausili E, et al. Relationship Between the Clinical-Neurophysiologic Pattern, Disability, and Quality of Life in Adolescents With Spina Bifida. J Child Neurol 2004; 19: 952-957

94. Roizen NJ, Higgins AM, Antshel KM, et al. 22q11.2 Deletion Syndrome: Are Motor Deficits More Than Expected for IQ Level? J Pediatr 2010; 157: 658-661.

95. Roy MW, Gilmore R, Walsh JW. Evaluation of children and young adults with tethered spinal cord syndrome. Utility of spinal and scalp recorded somatosensory evoked potentials. Surg Neurol 1986; 26: 241-248.

96. Salih MAM, Ahlsten G, Stalberg E, et al. Friedreich's ataxia in 13 children: Presentation and evolution with neurophysiologic, electrocardiographic, and echocardiographic features. J Child Neurol 1990; 5: 321-326.

97. Saroyan JM, Winfree CJ, Schechter WS, et al. Sciatic neuropathy after lower-extremity trauma - Successful treatment of an uncommon pain and disability syndrome in an adolescent. Am J Phys Med Rehabil 2007; 86: 597-600.

98. Saxena A, Masilamani K, Hartley L. Is the genetic basis of CMT predictable at the bedside? Dev Med Child Neurol 2011; 53: 43.

99. Tullu MS, Patil DP, Muranjan MN, et al. Human Immunodeficiency Virus (HIV) Infection in a Child Presenting as Acute Disseminated Encephalomyelitis. J Child Neurol 2011; 26: 99-102.

100. Udayashankar C, Oudeacoumar P, Nath A. Congenital insensitivity to pain and anhidrosis: A case report from South India. Indian J Dermatol 2012; 57: 503

101. Wan KS, Weng WC. Eosinophilic meningitis in a child raising snails as pets. Acta Trop 2004; 90: 51-53

102. Wang H, Cheng J, Xiao H, et al. Adolescent lumbar disc herniation: Experience from a large minimally invasive treatment centre for lumbar degenerative disease in Chongqing, China. Clin Neurol Neurosurg 2013; 115: 1415-1419.

103. Yagev R, Levy J, Shorer Z, Lifshitz T. Congenital insensitivity to pain with anhidrosis: ocular and systemic manifestations. Am J Ophthalmol 1999; 127: 322-326.

104. Ziv T, Watemberg N, Constantini S, Lerman-Sagie T. Cauda equina syndrome due to lumbosacral arachnoid cysts in children. Eur J Paediatr Neurol 1999; 3: 281-284.

105. Kroksmark AK, Beckung E, Tulinius M. Muscle strength and motor function in children and adolescents with spinal muscular atrophy II and III. Eur J Paediatr Neurol 2001; 5: 191-198.

106. Scott OM, Hyde SA, Vrbová G, Dubowitz V. Therapeutic possibilities of chronic low frequency electrical stimulation in children with Duchenne muscular dystrophy. J Neurol Sci 1990; 95: 171-182.

107. Van Der Linden ML, Hazlewood ME, Aitchison AM, et al. Electrical stimulation of gluteus maximus in children with cerebral palsy: effects on gait characteristics and muscle strength. Dev Med Child Neurol 2003; 45: 385-390

108. Brouwer B, Davidson LK, Olney SJ. Serial casting in idiopathic toe-walkers and children with spastic cerebral palsy. J Pediatr Orthop 2000; 20: 221-225.

109. Fowler EG, Knutson LM, DeMuth SK, et al. Paediatric Endurance and Limb Strengthening (PEDALS) for Children With Cerebral Palsy Using Stationary Cycling: A Randomized Controlled Trial. Phys Ther 2010; 90: 367-381.

110. Refshauge KM, Raymond J, Nicholson G, Dolder PA. Night splinting does not increase ankle range of motion in people with Charcot-Marie-Tooth disease: a randomised, cross-over trial. Aust J Physiother 2006; 52: 193-199.

111. Seniorou M, Thompson N, Harrington M, Theologis T. Recovery of muscle strength following multi-level orthopaedic surgery in diplegic cerebral palsy. Gait Posture 2007; 26: 475-481.

112. Hoesl M, Bruinink L, Harlaar J, Houdijk H. Evaluating gait adaptability in adolescents with CP - A treadmill approach. Gait Posture. 2012; 36: S98.

113. Liao HF, Liu YC, Liu WY, Lin YT. Effectiveness of loaded sit-to-stand resistance exercise for children with mild spastic diplegia: a randomized clinical trial. Arch Phys Med Rehabil 2007; 88: 25-31.

114. Lidbeck C, Tedroff K, Bartonek. Muscle strength does not explain standing ability in children with bilateral spastic cerebral palsy: A cross sectional descriptive study. BMC Neurol 2015; 15: 1.

115. Manzur AY, Hyde SA, Rodillo E, et al. A randomized controlled trial of early surgery in Duchenne muscular dystrophy. Neuromuscul Disord 1992; 2: 379-387.

116. Pagliano E, Moroni I, Baranello G, et al. Outcome measures for Charcot-Marie-Tooth disease: Clinical and neurofunctional assessment in children. J Peripher Nerv Syst 2011; 16: 237-242.

117. Reyes ML, Hernández M, Holmgren LJ, et al. High-frequency, low-intensity vibrations increase bone mass and muscle strength in upper limbs, improving autonomy in disabled children. J Bone Miner Res 2011; 26: 1759-1766.

118. Rose KJ, Burns J, North KN. Factors associated with foot and ankle strength in healthy preschool-age children and age-matched cases of Charcot-Marie-Tooth disease type 1A. J Child Neurol 2010; 25: 463-468.

119. Schoenmakers M, de Groot JF, Gorter JW, et al. Muscle strength, aerobic capacity and physical activity in independent ambulating children with lumbosacral spina bifida. Disabil Rehabil 2009; 31: 259-266.

120. Scholtes VA, Becher JG, Comuth A, et al. Effectiveness of functional progressive resistance exercise strength training on muscle strength and mobility in children with cerebral palsy: a randomized controlled trial. Dev Med Child Neurol 2010; 52: e107-e113

121. Scholtes VA, Dallmeijer AJ, Rameckers EA, et al. Lower limb strength training in children with cerebral palsy--a randomized controlled trial protocol for functional strength training based on progressive resistance exercise principles. BMC Pediatr 2008; 8: 1

122. Tedla JS. Strength training effects on balance in spastic diplegia subjects: A randomized controlled trial. J Pediatr Neurol 2014; 12: 15-28.

123. Verschuren O, Ketelaar M, Takken T, et al. Exercise programs for children with cerebral palsy: A systematic review of the literature. Am J Phys Med Rehabil 2008; 87: 404-417.

124. Arpin DJ, Stuberg W, Stergiou N, Kurz MJ. Motor control of the lower extremity musculature in children with cerebral palsy. Res Dev Disabil. 2013; 34: 1134-1143.

125. Chen CL, Chen CY, Liaw MY, et al. Efficacy of home-based virtual cycling training on bone mineral density in ambulatory children with cerebral palsy. Osteoporos Int 2013; 24: 1399-1406.

126. Ayalon M, Ben‐Sira D, Hutzler Y, Gilad T. Reliability of isokinetic strength measurements of the knee in children with cerebral palsy. Dev Med Child Neurol. 2000;42(6):398-402.

127. Berg‐Emons RJ, Baak MA, Barbanson DC, et al. Reliability of tests to determine peak aerobic power, anaerobic power and isokinetic muscle strength in children with spastic cerebral palsy. Dev Med Child Neurol 1996; 38: 1117-1125.

128. Engsberg JR, Ross SA, Collins DR. Increasing ankle strength to improve gait and function in children with cerebral palsy: a pilot study. Pediatr Phys Ther 2006; 18: 266-275.

129. Brown JK, Rodda J, Walsh EG, Wright GW. Neurophysiology of lower-limb function in hemiplegic children. Dev Med Child Neurol 1991; 33: 1037-1047.

130. Geerdink N, Cuppen I, Rotteveel J, et al. Contribution of the Corticospinal Tract to Motor Impairment in Spina Bifida. Pediatr Neurol 2012; 47: 270-278.

131. Gualdi S, Coluccini M, Carraro E, et al. Short term outcome effects of selective dorsal rhizotomy in patients with cerebral palsy. Gait Posture 2011; 33: S21-S22

132. Haberlová J, Seeman P. Utility of Charcot-Marie-Tooth neuropathy score in children with type 1A disease. Pediatr Neurol 2010; 43: 407-410.

133. Huddleston LM, Cohen R, Mercurio KJ. Unexpected diagnosis of charcot-marie-tooth in a 14-year-old girl with traumatic heel fracture: A case report. PM R. 2010; 2: S148.

134. Tshala-Katumbay D, Eeg-olofsson KE, Tylleskär T, Kazadi-Kayembe T. Impairments, disabilities and handicap pattern in konzo - A non-progressive spastic para/tetraparesis of acute onset. Disabil Rehabil 2001; 23: 731-736.

135. Kaya PDLDAL, Alemdaroʇlu I, Yilmaz O, et al. Effect of muscle weakness distribution on balance in neuromuscular disease. Pediatr Int 2015; 57: 92-97.

136. Komarowska M, Debek W, Wojnar JA, et al. Brown-Séquard syndrome in a 11-year-old girl due to penetrating glass injury to the thoracic spine. Eur J Orthop Surg Traumatol 2013; 23: S141-S143.

137. Labruyère R, Gerber CN, Birrer-Brütsch K, et al. Requirements for and impact of a serious game for neuro-paediatric robot-assisted gait training. Res Dev Disabil 2013; 34: 3906-3915.

138. Langerak NG, du Toit J, Burger M, et al. Spastic diplegia in children with HIV encephalopathy: first description of gait and physical status. Dev Med Child Neurol 2014; 56: 686-694.

139. Lin JP, Brown JK. Peripheral and central mechanisms of hindfoot equinus in childhood hemiplegia. Dev Med Child Neurol 1992; 34: 949-965.

140. Uchikawa K, Liu M, Hanayama K, et al. Functional status and muscle strength in people with Duchenne muscular dystrophy living in the community. J Rehabil Med 2004; 36: 124-129.

141. Wehby MC, O'Hollaren PS, Abtin K, et al. Occult tight filum terminale syndrome: results of surgical untethering. Pediatr Neurosurg 2004; 40: 51-57.

142. Furlan JC, Fehlings MG, Tator CH, Davis AM. Motor and Sensory Assessment of Patients in Clinical Trials for Pharmacological Therapy of Acute Spinal Cord Injury: Psychometric Properties of the ASIA Standards. J Neurotrauma 2008; 25: 1273-1301.

143. Pierce SR, Johnston TE, Shewokis PA, Lauer RT. Examination of spasticity of the knee flexors and knee extensors using isokinetic dynamometry with electromyography and clinical scales in children with spinal cord injury. J Spinal Cord Med 2008; 31: 208-214.

144. Acsadi G, Shy R, Dias A, et al. Paediatric neuropathy scale (CMTPed) for charcot-marie-tooth disease (CMT). Ann Neurol 2010; 68: S136-S137.

145. Burns J, Finkel R, Acsadi G, et al. Development and validation of the charcot-marie-tooth disease paediatric scale. J Peripher Nerv Syst 2010; 15: 253.

146. Imataka G, Yamazaki G, Negishi M, et al. Brown-Sequard syndrome in an 8-year-old girl: A case report. Dokkyo J Med Sci 2006; 33: 139-141.

147. Moody S, Mancias P. A genetic condition mimicking purely sensory CIDP. J Clin Neuromuscul Dis 2012; 13: 177-178
