# Supplementary material for: Clinimetric properties of lower limb neurological impairment tests for children and young people with a neurological condition: A systematic review
Source: PLoS One. 2017 Jul 3;12(7):e0180031. doi: 10.1371/journal.pone.0180031 (PMC5495217; doi:10.1371/journal.pone.0180031)
Supplement: S4 Table — (DOCX) [file pone.0180031.s004.docx]

**S4 Table. Characteristics of included clinimetric papers for neurological tests used on children and young people with a neurological condition.**

| **Neurological Test** | **Author** | **Participant characteristics** | | | | **Rater characteristics** | | **Measurement characteristics** | |
| --- | --- | --- | --- | --- | --- | --- | --- | --- | --- |
|  |  | ***n*** | **Age (years)** | **Sex** | **Diagnosis** | **Classification / Functional level** |  | |  |
| Handheld dynamometer | Berry ^[46]^ | *15* | 7 - 17 (mean 11y8mo) | not reported | CP | Diplegia (n = 12), Quadriplegia (n=3), Walked unassisted (n = 3), walked with assistance (n = 10), walked with assistance holding hands (n = 2) | 1 physiotherapist (9 years of experience in paediatric CP, received training using HHD) | | 2 prospective measurement sessions, 4-14 days apart |
| CMT Paediatric Scale | Burns ^[45]^ | *8* | 5 - 15 | 1 male, 7 female | CMT | CMT1A (n=4), CMT1B (n=2), CMT1E (n=1), CMT2A (n=1) | 8 clinicians: 3 physiotherapists, 1 neurologist, 1 paediatrician, 1 podiatrist, 1 occupational therapist, 1 medical graduate | | 1 prospective measurement session across 2 days with 2.5 hours between assessments on same day |
| Hand held dynamometer | Crompton ^[47]^ | *23* | 6 -14 (mean 9y6mo) | 14 male, 9 female | CP | GMFCS I (n=10), GMFCS II (n=9), GMFCS III (n=4) | 2 physiotherapists | | 2 prospective measurement sessions, 1 week apart |
| Hand held dynamometer | Effgen ^[48]^ | *12* | 10 – 17 | 3 male, 9 female | SB |  | 1 physiotherapist (experience not reported) | | 2 prospective measurement sessions, 23 days apart |
| Richmond Quantitative Measurement System | Escolar ^[55]^ | *12* | 6 – 14 (mean 9y4mo) | 12 male | DMD (n =7), LGMD (n=3), BMD (n = 2) |  | 12 physiotherapists (6+ years experience with paediatric neurological, trained by physiotherapist with 10+ experience in MMT) | | 4 prospective sessions over two days (3 hr break in between) |
| Manual Muscle Testing | Florence ^[56]^ | *102* | 5 – 15 | 102 male | DMD |  | 4 physiotherapists (16 – 20 years experience) | | 4 prospective measurements, 5 days apart initially and then 6 and 12 months |
| Manual Muscle Testing and Hand held dynamometry | Mahony ^[49]^ | *20* | 5 – 15 (mean 9y10mo) | 10 male, 10 female | SB | 13 walked unassisted, 3 with AFOs, 4 used wheelchairs | 2 physiotherapists (10+ years of experience with MMT, none with HHD), 1 physiotherapy student | | 1 prospective measurement session (15-20min) |
| ASIA Impairment Scale | Mulcahey ^[57]^ | *48* | 4 – 15 | Unknown | SCI (n = 48) | Incomplete tetraplegia (n=4), Complete tetraplegia (n=9), Incomplete paraplegia (n=4), Complete paraplegia (n=27) | 1 clinician (10+ years of experience with SCI) | | 2 prospective measurement sessions (24-48 hrs) |
| Hand held dynamometer | Stuberg ^[50]^ | *14* | 6 – 14 (mean 11y4mo) | 14 male | DMD | 6 walked unassisted (Vignos classification^a^ 1 (n = 2), 3 (n = 1), 4 (n = 3)), 2 with AFOs (Vignos 7 (n = 2)), 6 unable to walk (Vignos 8 (n = 4), 9 (n = 2)) | 1 physiotherapist (author) | | 2 prospective measurement sessions, 1 day apart |
| Hand held dynamometer | Taylor ^[51]^ | *10* | 8 – 18 (mean 13y6mo) | 6 male, 4 female | CP | GMFCS 1 (n=5), II (n =3), III (n=2) | 1 physiotherapist (experienced in musculoskeletal assessment) | | 2 prospective measurement sessions, 6 weeks apart |
| Hand held dynamometer and Standing heel rise | Van Vulpen ^[52]^ | *20* | 4 – 6 (Mean 4y11mo)  6 – 10y8mo (mean 8y2mo) | 1 male, 9 female (3-5y group)  5 male, 5 female (6-10y group) | CP | GMFCS I (n = 13), GMFCS II (n = 7) | 1 physiotherapist (15 years of experience in paediatric CP and trained in HHD) | | 2 prospective measurement sessions, within 3 weeks apart |
| Hand held dynamometry | Verschuren ^[53]^ | *25* | 8 -17 (mean 11y11mo), 7 - 17 (mean 10y11mo) | 15 male, 10 female | CP | GMFCS I (n = 14), GMFCS II (n = 11) | 2 physiotherapists (authors) (Previously trained in HHD) | | 1 prospective measurement session, 10 minute break between examiners |
| Hand held dynamometry | Williemse ^[54]^ | *14* | 7 – 13 (mean 10y2mo) | 9 male, 5 female | CP | Hemiplegic (n=6), Diplopic (n=8)  GMFCS I (n=8), II (n=3), III (n=3) | 1 physiotherapist (received training consisting of instructions on how to use HHD, apply standard test position and stabilization). | | 2 prospective measurement sessions, 2-5 days apart |
|  |  |  |  |  |  |  |  | |  |

HHD, Hand held dynamometer; MMT, Manual Muscle Test; CP, Cerebral Palsy, DMD, Duchenne’s Muscular Dystrophy; CMT, Charcot-Marie Tooth; SB, Spina Bifida; GMFCS, Gross Motor Functional Classification Scale

^a^ Vignos classification as cited in Stuberg et al. ^[50]^ 1 = Walks and climbs stairs without assistance, 2 = walks and climbs stairs with aid of railing, 3 = walks independently and climbs stairs slowly with rail, 4 = walks independently and rises from chair unassisted but cannot climb stairs, 5 = walks independently in bilateral knee-ankle-foot-orthoses, 6 = walks independently in bilateral knee-ankle-foot orthoses, 7 = walks with orthoses and assistance of one person, 8 = stands in orthoses but is unable to walk even with assistance, 9 = wheelchair use for mobility

45. Burns J, Ouvrier R, Estilow T, et al. Validation of the Charcot-Marie-Tooth disease paediatric scale as an outcome measure of disability. Ann Neurol 2012; 71: 642-652.

46. Berry ET. Intrasession and intersession reliability of handheld dynamometry in children with cerebral palsy. Pediatr Phys Ther 2004; 16: 191-198.

47. Crompton J, Galea MP, Phillips B. Hand-held dynamometry for muscle strength measurement in children with cerebral palsy. Dev Med Child Neurol 2007; 49: 106-111

48. Effgen SK, Brown DA. Long-term stability of hand-held dynamometric measurements in children who have myelomeningocele. Phys Ther 1992; 72: 458-465.

49. Mahony K, Hunt A, Daley D, et al. Inter-tester reliability and precision of manual muscle testing and hand-held dynamometry in lower limb muscles of children with spina bifida. Phys Occup Ther Pediatr 2009; 29: 44-59.

50. Stuberg WA, Metcalf WK. Reliability of quantitative muscle testing in healthy children and in children with Duchenne muscular dystrophy using a hand-held dynamometer. Phys Ther 1988; 68: 977-982.

51. Taylor NF, Dodd KJ, Graham HK. Test-retest reliability of hand-held dynamometric strength testing in young people with cerebral palsy. Arch Phys Med Rehabil 2004; 85: 77-80.

52. Van Vulpen LF, De Groot S, Becher JG, et al. Feasibility and test-retest reliability of measuring lower-limb strength in young children with cerebral palsy. Eur J Phys Rehab Med 2013; 49: 803-813.

53. Verschuren O, Ketelaar M, Takken T, et al. Reliability of hand-held dynamometry and functional strength tests for the lower extremity in children with Cerebral Palsy. Disabil Rehabil 2008; 30: 1358-1366.

54. Willemse L, Brehm MA, Scholtes VA, et al. Reliability of isometric lower-extremity muscle strength measurements in children with cerebral palsy: implications for measurement design. Phys Ther 2013; 93: 935-941.

55. Escolar D, Henricson E, Mayhew J, et al. Clinical evaluator reliability for quantitative and manual muscle testing measures of strength in children. Muscle Nerve 2001; 24: 787-793.

56. Florence JM, Pandya S, King WM, et al. Intrarater reliability of manual muscle test (Medical Research Council scale) grades in Duchenne's muscular dystrophy. Phys Ther 1992; 72: 115-122.

57. Mulcahey M, Gaughan J, Betz R, Johansen K. The International Standards for Neurological Classification of Spinal Cord Injury: reliability of data when applied to children and youths. Spinal Cord. 2007;45:452-459
